# Cerebrospinal Fluid Profile of Lipid Mediators in Alzheimer’s Disease

**DOI:** 10.1007/s10571-022-01216-5

**Published:** 2022-04-01

**Authors:** Khanh V. Do, Erik Hjorth, Ying Wang, Bokkyoo Jun, Marie-Audrey I. Kautzmann, Makiko Ohshima, Maria Eriksdotter, Marianne Schultzberg, Nicolas G. Bazan

**Affiliations:** 1 Neuroscience Center of Excellence, School of Medicine, Louisiana State University Health New Orleans, 2020 Gravier Street, Suite D, New Orleans, LA 70112 USA; 2 Division of Neurogeriatrics, Department of Neurobiology, Care Sciences and Society, Karolinska Institutet, BioClinicum J9:20, Visionsgatan 4, 171 64 Solna, Sweden; 3 Division of Clinical Geriatrics, Department of Neurobiology, Care Sciences and Society, Karolinska Institutet, Karolinska University Hospital, 141 86 Huddinge, Sweden; 4 Present Address: Faculty of Medicine, PHENIKAA University, Hanoi, 12116 Vietnam; 5 Present Address: PHENIKAA Research and Technology Institute (PRATI), A&A Green Phoenix Group JSC,, No.167 Hoang Ngan, Trung Hoa, Cau Giay, Hanoi, 11313 Vietnam

**Keywords:** β-amyloid, Tau, Biomarker, Gender, Inflammation, Resolution, Subjective cognitive impairment, Cognitive tests

## Abstract

Alzheimer’s disease (AD) develops into dementia over a period of several years, during which subjective cognitive impairment (SCI) and mild cognitive impairment (MCI) can be used as intermediary diagnoses of increasing severity. Chronic neuroinflammation resulting from insufficient resolution is involved in the pathogenesis of AD and is associated with cognitive impairment. Specialized pro-resolving lipid mediators (LMs) that promote the resolution of inflammation may be valuable markers in AD diagnosis and as therapeutic targets. Liquid chromatography–tandem mass spectrometry was used to analyze pro-resolving and pro-inflammatory LMs in cerebrospinal fluid (CSF) from patients with cognitive impairment ranging from subjective impairment to a diagnosis of AD and correlated to cognition, CSF tau, and β-amyloid. Resolvin (Rv) D4, RvD1, neuroprotectin D1 (NPD1), maresin 1 (MaR1), and RvE4 were lower in AD and/or MCI compared to SCI. The pro-inflammatory LTB_4_ and 15-HETE were higher in AD and MCI, respectively, while PGD2, PGE2, and PGF2a were decreased in AD, compared to SCI. RvD4 was also negatively correlated to AD tangle biomarkers, and positive correlations to cognitive test scores were observed for both pro-resolving LMs and their precursor fatty acids. In this exploratory study of the lipidome in CSF of AD, MCI, and SCI, the results indicate a shift in the LM profile from pro-resolving to pro-inflammatory in progression to AD, suggesting that it may be of use as a biomarker when followed by confirmation by replication studies.

**Graphical Abstract:**

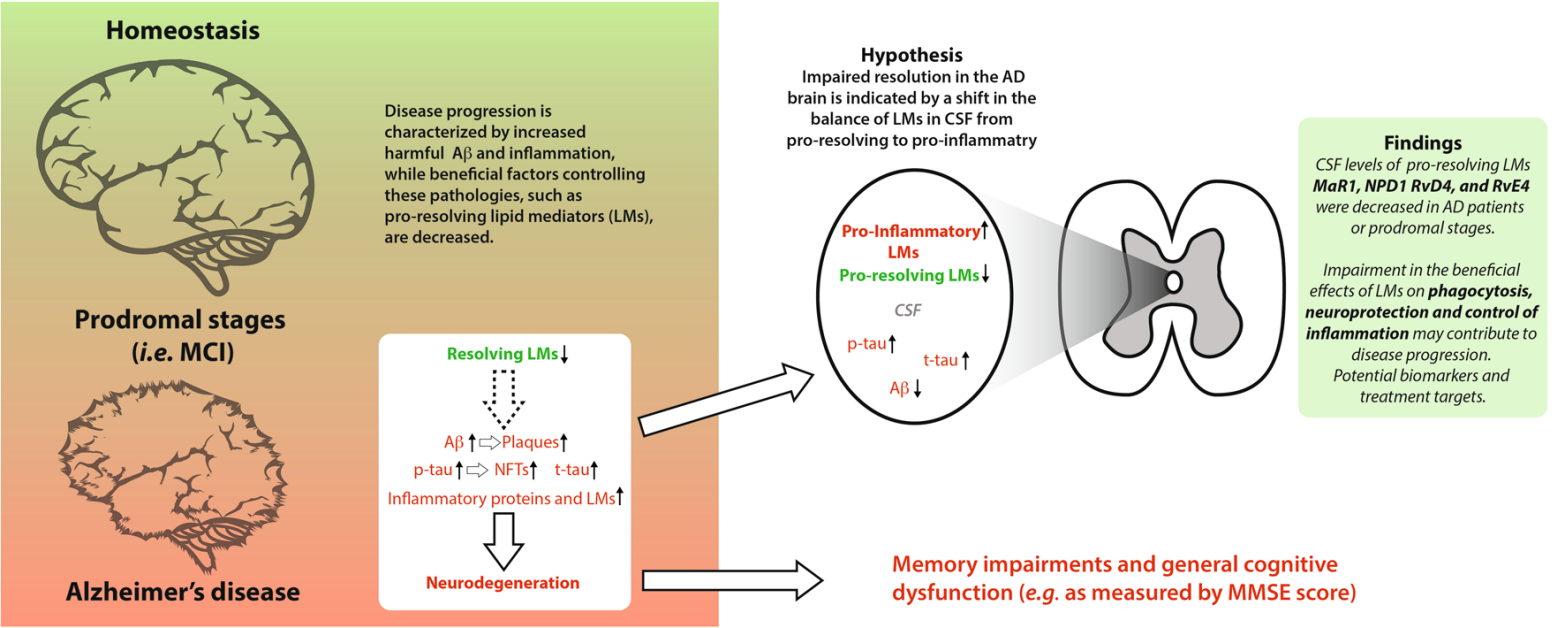

**Supplementary Information:**

The online version contains supplementary material available at 10.1007/s10571-022-01216-5.

## Introduction

Alzheimer’s disease (AD) is the most common dementia in the aged population (Prince et al. 2013; Alzheimer’s Association 2021). The disease progression is insidious and takes over decades to develop dementia. The pathology in the AD brain includes neuronal and synapse loss, widespread deposits of senile plaques consisting of β-amyloid (Aβ) peptide and neurofibrillary tangles of phosphorylated (p)-tau protein, and activated microglia (Maccioni et al. 2001; Heneka et al. 2015; Scheltens et al. 2016). A commonly used nomenclature of increasing severity starts with subjective cognitive impairment (SCI) (Reisberg et al. 2008), then mild cognitive impairment (MCI) (Albert et al. 2011), and finally, dementia due to AD (Jack et al. 2018). The mini-mental state examination (MMSE) is a widely used test of cognitive function, and the severity of AD is commonly assessed by MMSE (Arevalo-Rodriguez et al. 2015), although other tests are increasingly used, such as Montreal cognitive assessment (MoCA) (Luis et al. 2009). Since the diagnosis of SCI is based on the individual experiences of memory problems, clinical assessments, including MMSE scores, are within the normal range (Reisberg et al. 2008), while MCI is diagnosed as decreased cognitive function with minimal or no functional decline (Albert et al. 2011). In severe dementia, not only cognitive and mental functions, but also communication ability and mobility are impaired (Winblad et al. 2004).

Molecular biomarkers in cerebrospinal fluid (CSF) of Aβ and tau pathology are used to facilitate AD diagnosis (Olsson et al. 2016). However, AD is a heterogeneous and multifactorial disease and should be regarded from a broader perspective than from only Aβ and p-tau (Lue et al. 1996). It is important to expand the range of molecular factors used as biomarkers reflecting other mechanisms of the pathogenesis, such as inflammation and metabolic alterations. Inflammatory responses in AD are well known (McGeer and McGeer 1995; Heneka et al. 2015), and there is accumulating evidence of its occurrence early in the disease process (Rodriguez-Vieitez et al. 2016). Aside from being regulated by cytokines and chemokines, inflammation engages a prominent network of lipid mediators (LMs) with well-known bioactivities, such as the fever response (Coceani et al. 1986; Kozak and Fraifeld 2004) and pain (Juan 1978). LMs also play an essential role in the resolution of inflammation and the initiation of tissue restoration, an active process regulated by specialized pro-resolving LMs. These include the lipoxins (LX), protectins (PD), resolvins (Rv), and maresins (MaR), which are derived from the omega-3 and -6 polyunsaturated fatty acids (PUFAs) docosahexaenoic acid (DHA), arachidonic acid (AA), and eicosapentaenoic acid (EPA) (Buckley et al. 2014; Serhan et al. 2014). Although it is a relatively new field of research, studies on various pathologies revealed LM involvement (Gonzalez-Gay et al. 2008).

AD brains demonstrate lower levels of pro-resolving LMs than healthy controls (Lukiw et al. 2005; Wang et al. 2015; Zhu et al. 2016), while the expression of their receptors is increased (Wang et al. 2015; Emre et al. 2020). In vitro studies demonstrate that pro-resolving LMs improve cell survival, reduce Aβ production in neuronal models (Lukiw et al. 2005; Medeiros et al. 2013; Dunn et al. 2015; Zhu et al. 2016; Lee et al. 2020; Wang et al. 2021), and down-regulate inflammation and increase Aβ phagocytosis in glia (Lukiw et al. 2005; Medeiros et al. 2013; Dunn et al. 2015; Zhu et al. 2016; Lee et al. 2020; Wang et al. 2021). Reduction of AD pathologies and attenuation of cognitive impairment (Medeiros et al. 2013; Dunn et al. 2015; Kantarci et al. 2018; Yin et al. 2019; Lee et al. 2020; Emre et al. 2022) have been shown in in vivo models. To pave the way for future treatments and biomarkers based on the resolution of inflammation, we aimed at analyzing the pro-inflammatory and pro-resolving lipidome in CSF in cohorts of AD, MCI, or SCI patients and how the lipidome is associated with cognitive dysfunction and biomarkers of plaques and tangles. In view of gender differences in the prevalence of AD and lipid metabolism, we addressed gender-dependent alterations of lipidome in relation to cognitive impairment.

## Materials and Methods

### Recruitment of Study Subjects

**Fig. 1:**
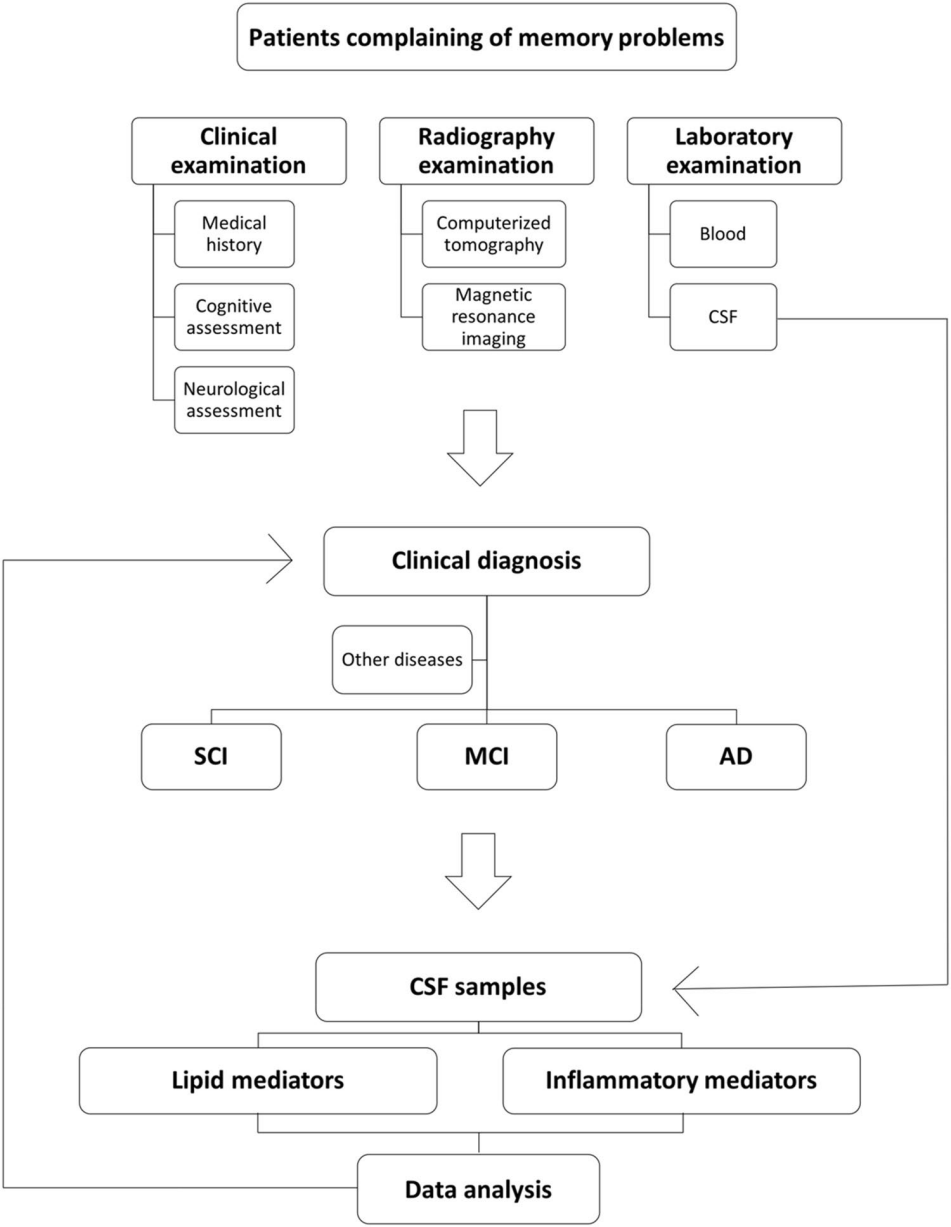
Study flow-chart. Cerebrospinal fluid (CSF) samples were obtained from a cohort of 136 patients subjected to clinical, radiography, and laboratory examinations for the diagnosis of subjective cognitive impairment (SCI) (*n* = 53), mild cognitive impairment (MCI) (*n* = 43), and Alzheimer's disease (AD) (*n* = 40)

The study population consisted of 136 participants with SCI (*n* = 53; 33 female and 20 male), MCI (*n* = 43; 23 female and 20 male), or AD (*n* = 40; 24 female and 16 male) from the Memory Clinic at Karolinska University Hospital, Huddinge, Sweden. All participants gave informed consent and agreed to donate their CSF to the Gedoc biobank for scientific research. The study was approved by the Regional Human Ethics Committee of Stockholm (2011/680-31, 2014/1921-32, and 2020-02023). Table 1 lists the demographics of the study population. The recruitment procedure details are outlined in Fig. 1. Data on age, gender, cognition, CSF AD biomarkers [Aβ_42_, total (t)-tau and phosphorylated (p)-tau], the mini-mental state examination (MMSE) test (Folstein et al. 1975), and clinical diagnosis were retrieved from the biobank database at the clinic. The CSF biomarker levels were determined by ELISAs (INNOTEST®, Innogenetics, Ghent, Belgium) with the following cut-off values indicating pathology: Aβ_42_ < 550 pg/mL, t-tau > 400 pg/mL, and p-tau > 80 pg/mL. The ICD-10 criteria were used for AD diagnosis (Naik and Nygaard 2008), and the Winblad criteria were used for the diagnosis of MCI (Winblad et al. 2004). A diagnosis of SCI was established when clinical tests did not indicate pathology (Reisberg et al. 2008).

**Table 1:** Cohort characteristics

Entire cohort			
	AD	MCI	SCI
	*n* = 40	*n* = 43	*n* = 53
	(*F* = 24, *M* = 16)	(*F* = 23, *M* = 20)	(*F* = 33, *M* = 20)
	Median ± SEM	Median ± SEM	Median ± SEM
Age (y)	78.5 ± 1.3	66 ± 1.3	64 ± 1
MMSE	24 ± 0.5	28 ± 0.2	29 ± 0.2
Aβ_42_	434.5 ± 18.1	851 ± 34.2	909 ± 24
t-tau	523 ± 48.8	269 ± 28.9	260 ± 12.8
p-tau	59 ± 4.4	40 ± 3	37 ± 1.6

Age-matched sub-cohort			
	AD	MCI	SCI
	*n* = 15	*n* = 17	*n* = 21
	(*F* = 6, *M* = 9)	(*F* = 8, *M* = 9)	(*F* = 12, *M* = 9)
	Median ± SEM	Median ± SEM	Median ± SEM
Age (y)	66 ± 9.2	66 ± 2.7	66 ± 6.6
MMSE	24 ± 2.8	28 ± 1.5	29 ± 1.4
Aβ_42_	421 ± 124.4	831 ± 195.2	900 ± 183.3
t-tau	545 ± 335.6	300 ± 199.3	261 ± 97.2
p-tau	59 ± 28.5	41 ± 18.8	39 ± 12.1

Data are described as median with interquartile (Q) range in pg/mL for amyloid β_42_ (Aβ_42_), total tau (t-tau), and phosphorylated tau (p-tau)

*AD* Alzheimer’s disease, *CSF* cerebrospinal fluid, *F* female, *M* male, *MCI* mild cognitive impairment, *MMSE* mini-mental state examination, *SCI* subjective cognitive impairment, *y* years

The clinical data were collected on the same or adjacent day of the lumbar puncture, and the CSF aliquots were kept at − 80 °C until used. Prior to analysis, aliquots were thawed on ice.

### Analysis of Lipid Mediators

Liquid chromatography with tandem mass spectrometry (LC-MS/MS) was used to assess a total of 22 lipids in the CSF samples, including pro-resolving LMs [LXA_4_, MaR1, MaR2, neuroprotectin D1 (NPD1), RvD1, RvD3, RvD4, RvE1, and RvE4], pro-inflammatory LMs (leukotriene B_4_, LTB_4_), prostaglandins (PGD2, PGE2, and PGF2α), their precursors (EPA, AA, and DHA), and the intermediate products in the metabolic pathways [14-hydroxy-docosahexaenoic acid (14-HDHA), 17-HDHA, 20-HDHA, 12-hydroxyeicosatetraenoic (12-HETE), 14-HETE, and 15-HETE] (Supplementary Fig. 1). Fatty acids were extracted from CSF samples using a liquid–liquid lipid extraction method based on chloroform–methanol extraction (Folch et al. 1957). Briefly, since the volume of CSF samples was small (< 700 μL), extraction was done by adding 9 ml of CHCl_3_/MeOH = 2:1. Internal standard mix (PGD2-d4, LTB4-d4, 15-HETE-d8, EPA-d5, and AA-d8) was added. Then 2 ml of pH3.5 H_2_O was added, the resulting upper aqueous phase discarded, and the bottom organic phase was dried down under a gentle N_2_ gas stream. The lipid extract was re-constituted in 50 μL of MeOH/H_2_O = 1:1 solvent and samples loaded onto a Xevo TQ-S equipped with Acquity I Class UPLC (Waters, Milford, MA, USA). Chromatographic separation was performed using CORTECS C18 2.7 μm column (4.6 × 100 mm; Waters, Milford, MA, USA). Initially, 56.2% of the mobile phase A (MeOH/H2O = 2:8, 0.01% AcA) gradually decreased to 25% for the first 8 min, then 3 min of isocratic run, followed by 100% B (MeOH, 0.01% AcA) at 18.1 min. The isocratic run of 100% B till 25 min is followed. Finally, it comes back to the initial condition for 5 min. The capillary voltage was − 2.5 kV, desolvation temperature at 600 °C, desolvation gas flow at 1100 L/Hr, cone gas at 150 L/Hr, and nebulizer pressure at 7.0 Bar with the source temperature at 150 °C.

### Statistical Analysis

To investigate differences between diagnostic groups, Kruskal–Wallis was performed in Statistica v13 (Tibco, Palo Alto, USA), followed by Dunn’s *post hoc* test corrected for multiple comparisons. The association of LMs to cognition and AD biomarkers was tested with Spearman Rank Order Correlation in Statistica v13. A *P*-value of < 0.05 was considered statistically significant.

## Results

CSF samples from cases with different degrees of memory dysfunction according to objective tests (AD or MCI) or subjective memory complaints (SCI) were analyzed by LC-MS/MS with regard to bioactive LMs, their fatty acid precursors, and intermediate derivatives. The resulting data were subject to statistical analysis in the entire cohort, *i.e.*, including all cases, and in a sub-cohort, including cases from the AD, MCI, and SCI groups with similar age. The median detected level and interquartile range for each LM within the diagnostic groups are presented in Supplementary Tables 1 and 3.

### Differences in Lipids Between Diagnostic Groups

**Fig. 2:**
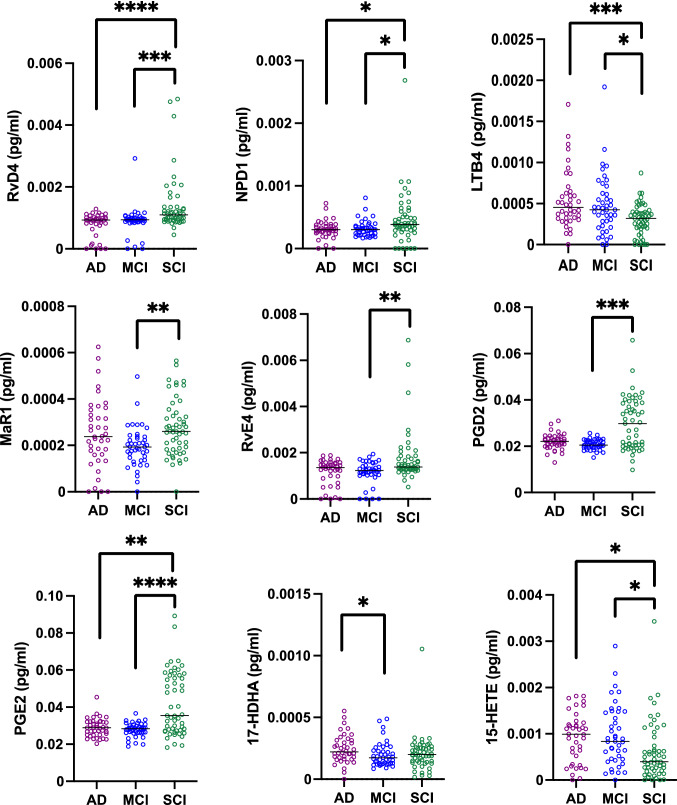
Pro-resolving LMs are reduced in CSF from MCI and AD patients, while pro-inflammatory LMs show a mixed pattern. Lipid mediators (LMs) were assessed in the cerebrospinal fluid (CSF) samples from patients with Alzheimer’s disease (AD) (*n* = 40), mild cognitive impairment (MCI) (*n* = 43), or subjective cognitive impairment (SCI) (*n* = 53), using liquid chromatography–tandem mass spectrometry (LC-MS/MS). The levels of resolvin (Rv) D4 (D4) and neuroprotectin D1 (NPD1) were reduced in CSF from AD and MCI compared to SCI, while the levels of the pro-inflammatory LM leukotriene B4 (LTB4) levels were higher in AD. The levels of maresin 1 (MaR1) and RvE4 were significantly lower in MCI patients compared to SCI. The levels of the intermediate precursor for RvD4, NPD1, and MaR1, 17-hydroxy docosahexaenoic acid (17-HDHA), were higher in AD than in MCI, and the levels of the intermediate precursor 15-hydroxyeicosatetraenoic acid (15-HETE) were lower in SCI compared to MCI and AD. The levels of prostaglandin (PG) D_2_ were lower in CSF from MCI patients compared with SCI, and the PGE_2_ levels were lower in AD and MCI patients compared to SCI. Comparisons between groups were performed by Kruskal–Wallis ANOVA with Dunn’s multiple comparisons post hoc test (*P < 0.05, **P < 0.005, ***P < 0.001, ****P < 0.0001)

**Fig. 3:**
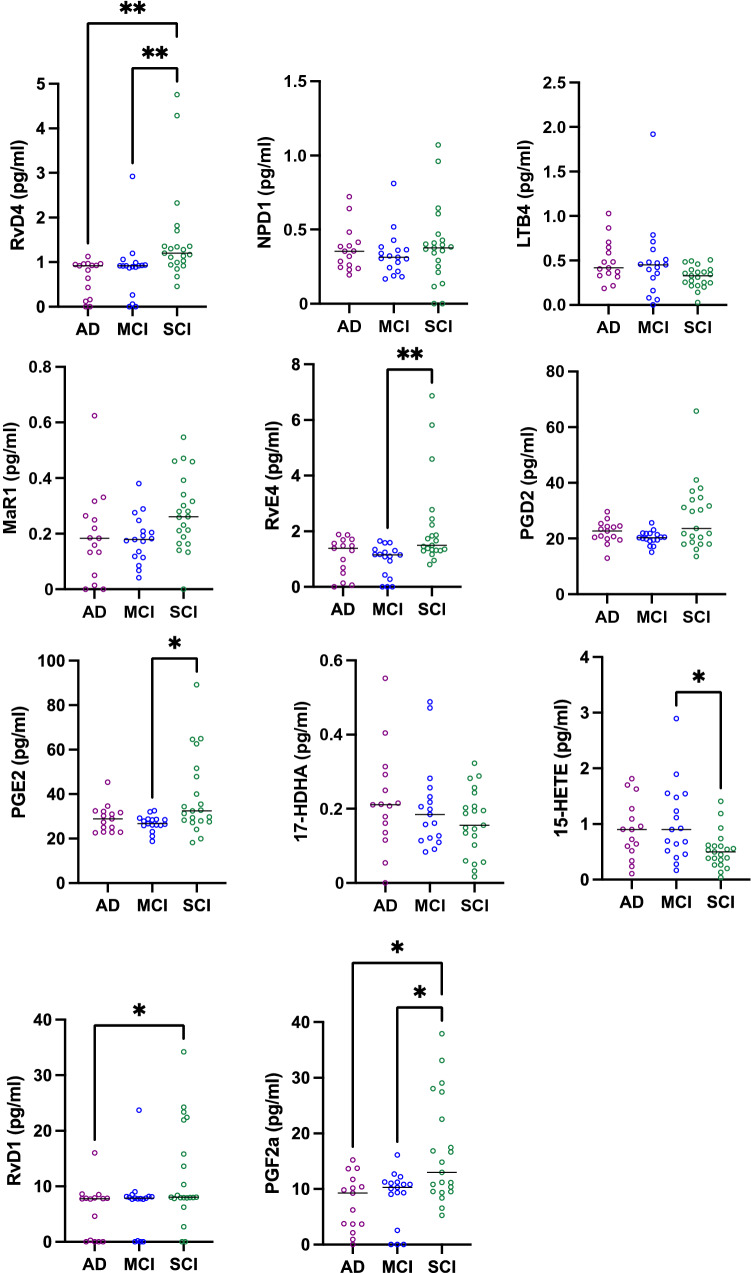
Pro-resolving LMs are reduced in CSF from MCI and AD patients in an age-matched sub-cohort. Lipid mediators (LMs) were assessed in the cerebrospinal fluid (CSF) samples from patients with Alzheimer’s disease (AD) (*n* = 15), mild cognitive impairment (MCI) (*n* = 17), or subjective cognitive impairment (SCI) (*n* = 21), using liquid chromatography–tandem mass spectrometry (LC-MS/MS). The reduced levels of resolvin (Rv) D4 in AD and MCI compared to SCI are confirmed in this smaller age-matched sub-cohort. Also, the reduced levels of RvE4 and prostaglandin (PG) E2 in MCI compared to SCI are confirmed, whereas there is no difference between AD and SCI for PGE2 in the age-matched cohort. Similarly, the increased levels of the intermediate precursor 15-hydroxyeicosatetraenoic acid (15-HETE) in MCI compared to SCI are confirmed, but not the difference between AD and SCI. The other alterations seen in the total cohort do not reach statistical significance in the age-matched cohort, but two additional changes were observed, *i.e.*, reduced levels of RvD1 in AD compared to SCI and of PGF2a in AD and MCI compared to SCI. Comparisons between groups were performed by Kruskal–Wallis ANOVA with Dunn’s multiple comparisons post hoc test (*P < 0.05, **P < 0.005)

Analysis of pro-resolving and pro-inflammatory LMs in CSF samples for all cases in the entire cohort showed that levels of the pro-resolving LMs RvD4 and NPD1 were lower in the AD (*P* < 0.00005 and *P* < 0.05, respectively) and MCI (*P* < 0.0005 and *P* < 0.05, respectively) group compared to the SCI group (Fig. 2), whereas levels of the pro-inflammatory LM LTB_4_ were higher in AD (*P* < 0.001) and MCI (*P* < 0.05) compared to SCI (Fig. 2). Analysis of the age-matched cohort showed the same results for RvD4, i.e., for AD (*P* < 0.005) and MCI (*P* < 0.005) compared to SCI (Fig. 3), whereas the differences between the diagnostic groups for NPD1 and LTB4 were not statistically significant. However, additional differences were found in the age-matched cohort, including a reduction in RvD1 in AD cases compared to SCI (*P* < 0.05; Fig. 3).

Comparing the diagnostic groups within the male and female group separately showed that for RvD4, there was a significant difference between AD and SCI for both women and men, but the difference between MCI and SCI was seen only in women (Supplementary Fig. 2). In the case of LTB4, higher levels in AD than in SCI were seen in women, whereas the increase in MCI compared with SCI reached statistical significance only in men (Supplementary Fig. 2). The differences seen for NPD1 were small and not seen upon analyzing male and female groups separately. On the other hand, the levels of RvE1 were reduced in men with AD compared to SCI (Supplementary Fig. 2), but not in women or when analyzing both men and women together. Similarly, the levels of RvD3 were lower in MCI compared to SCI in women (Supplementary Fig. 2), but not in men or in both groups together.

The pro-resolving LMs MaR1 (*P* < 0.005; Fig. 2) and RvE4 (*P* < 0.005; Fig. 2) were lower in CSF samples from MCI patients compared to SCI cases in the entire cohort. In the age-matched cohort, there was no difference observed for MaR1, but the decrease in RvE4 in the MCI patients remained (P < 0.005; Fig. 3). The difference in MaR1 seems to be due mainly to a difference in men (Supplementary Fig. 2), whereas this difference was not seen for women. Interestingly, the levels of the pro-inflammatory LXA_4_ were lower in men with MCI than in SCI (Supplementary Fig. 2), a finding not seen when analyzing men and women together in the entire or the age-matched cohort.

The levels of PGD_2_ (*P* < 0.0005) and PGE_2_ (*P* < 0.0001) were lower in MCI patients compared to SCI in the entire cohort (Fig. 2), and these differences were seen both in women and in men (Supplementary Fig. 2). In addition, the PGE_2_ levels were lower in AD compared to SCI (*P* < 0.005; Fig. 2), a difference also seen in women (Supplementary Fig. 2). In the age-matched cohort, there was no difference between the diagnostic groups for PGD2, but in the case of PGE2, there were still reduced levels in MCI compared to SCI (*P* < 0.05), whereas the difference between AD and SCI were not seen in the age-matched cohort (Fig. 3). However, additional differences were found in the age-matched cohort for PGF2a showing lower levels in AD compared to SCI (*P* < 0.05; Fig. 3).

The levels of the intermediate LM precursor 17-HDHA were higher in AD than in MCI (*P* < 0.05), and levels of 15-HETE were higher in AD (*P* < 0.01) and MCI (*P* < 0.01) than in SCI within the entire cohort (Fig. 2). The difference between AD and SCI reached statistical significance in women (Supplementary Fig. 2). In the age-matched cohort, there was no difference between the diagnostic groups for 17-HDHA, but there was still an increase in MCI compared to SCI for 15-HETE (*P* < 0.05), whereas the difference between AD and SCI was not seen in the age-matched cohort (Fig. 3).

Regarding the n-3 and n-6 PUFA precursors, AA, EPA, or DHA, there were no significant differences between the three diagnostic groups, in either the entire cohort or in the age-matched cohort. An exception was in the case of DHA, where the levels in men were lower in AD compared to SCI (Supplementary Fig. 2).

### Correlations to Cognitive Function, CSF Biomarkers of Plaque and Tangle Pathology

Correlative relationships were investigated using the Spearman rank-order test. The complete results from the analysis of correlations, including all LMs and PUFAs, can be seen in Supplementary Tables 2 (entire cohort) and 4 (age-matched cohort).

#### MMSE

Our analyses of correlations suggest that for several lipids, high levels are associated with less deterioration of cognition in AD cases as assessed by the MMSE test (Supplementary Tables 2a, 4a). Analysis of the entire cohort showed that the levels of RvD4 displayed the strongest positive correlation to MMSE score (*r* = 0.29, *P* < 0.001), and this correlation was confirmed in the age-matched cohort (*r* = 0.39, *P* < 0.005). Other lipids showing a positive correlation to MMSE when including all three diagnostic groups were DHA (*r* = 0.21), EPA (*r* = 0.18) and PGE_2_ (*r* = 0.18), and RvD1 (*r* = 0.17), all with a significance level of *P* < 0.05. Analysis of the age-matched cohort confirmed the correlation for DHA (*r* = 0.34, *P* < 0.05) but not for EPA. The correlations between MMSE and PGE2 and between MMSE and RvD1, respectively, were not seen in the age-matched cohort, but there was a positive correlation for PGF2a (*r* = 0.30, *P* < 0.05) and for RvE1 (*r* = 0.34, *P* < 0.05) in the latter cohort.

**Fig. 4:**
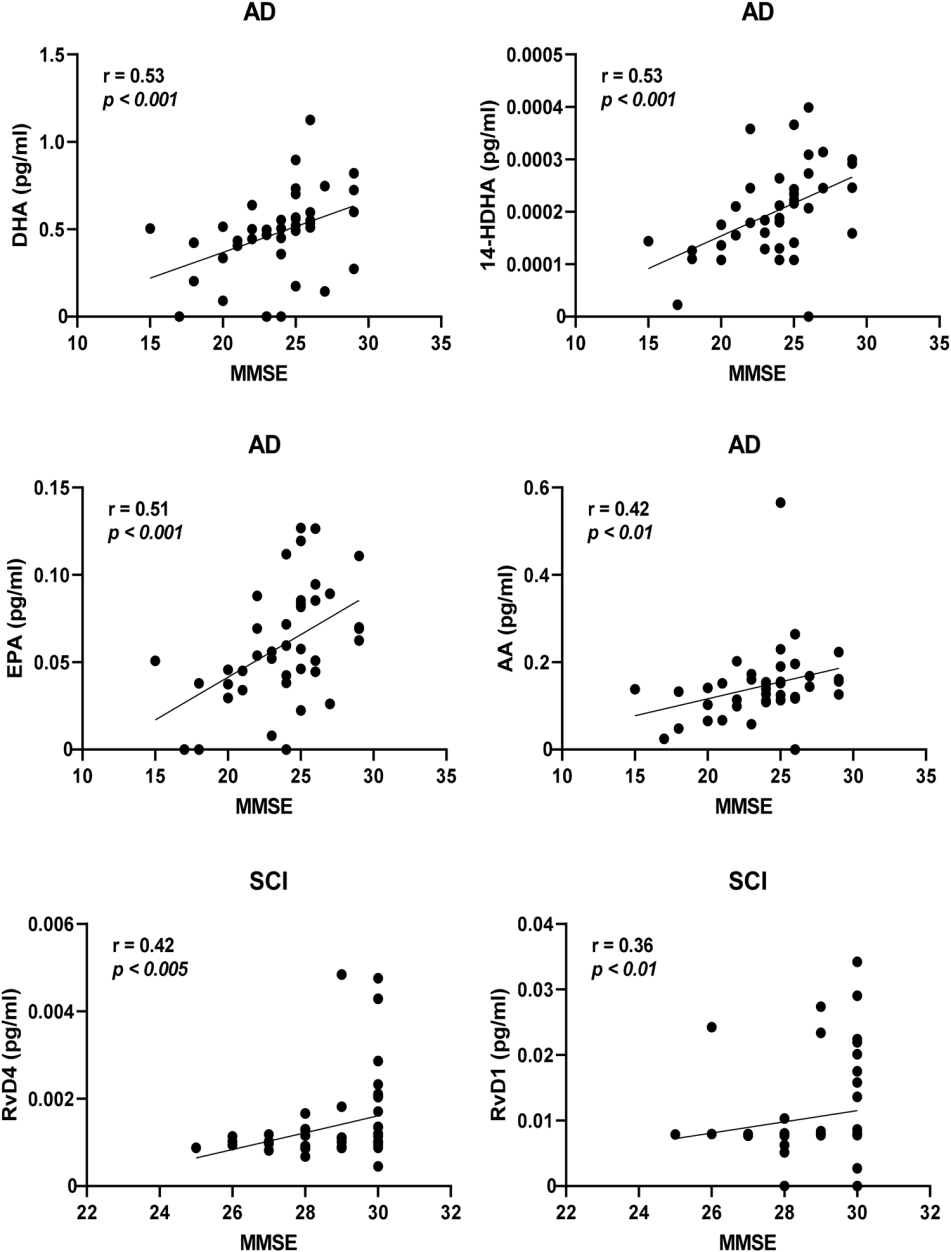
Low levels of bioactive LMs correlate with low test scores for cognitive function. The levels of lipid mediators (LMs) in cerebrospinal fluid (CSF) samples from the total cohort of patients with Alzheimer’s disease (AD) (*n* = 40), mild cognitive impairment (MCI) (*n* = 43), or subjective cognitive impairment (SCI) (*n* = 53) were correlated to the mini-mental state examination (MMSE) test scores and the r-value according to Spearman rank-order test is given together with the *P*-value. Resolvin (Rv) D1 and RvD4 show positive correlation to the MMSE scores in the SCI group and when analyzing all three diagnostic groups together (Supplementary Table 2a). The omega-3 fatty acids docosahexaenoic acid (DHA) and eicosapentaenoic acid (EPA) and the intermediate precursor 14-hydroxy-docosahexaenoic acid (14-HDHA) are positively correlated to the MMSE scores in the AD group. Also, levels of the omega-6 fatty acid arachidonic acid (AA) are positively correlated to the MMSE scores in the AD group

**Fig. 5:**
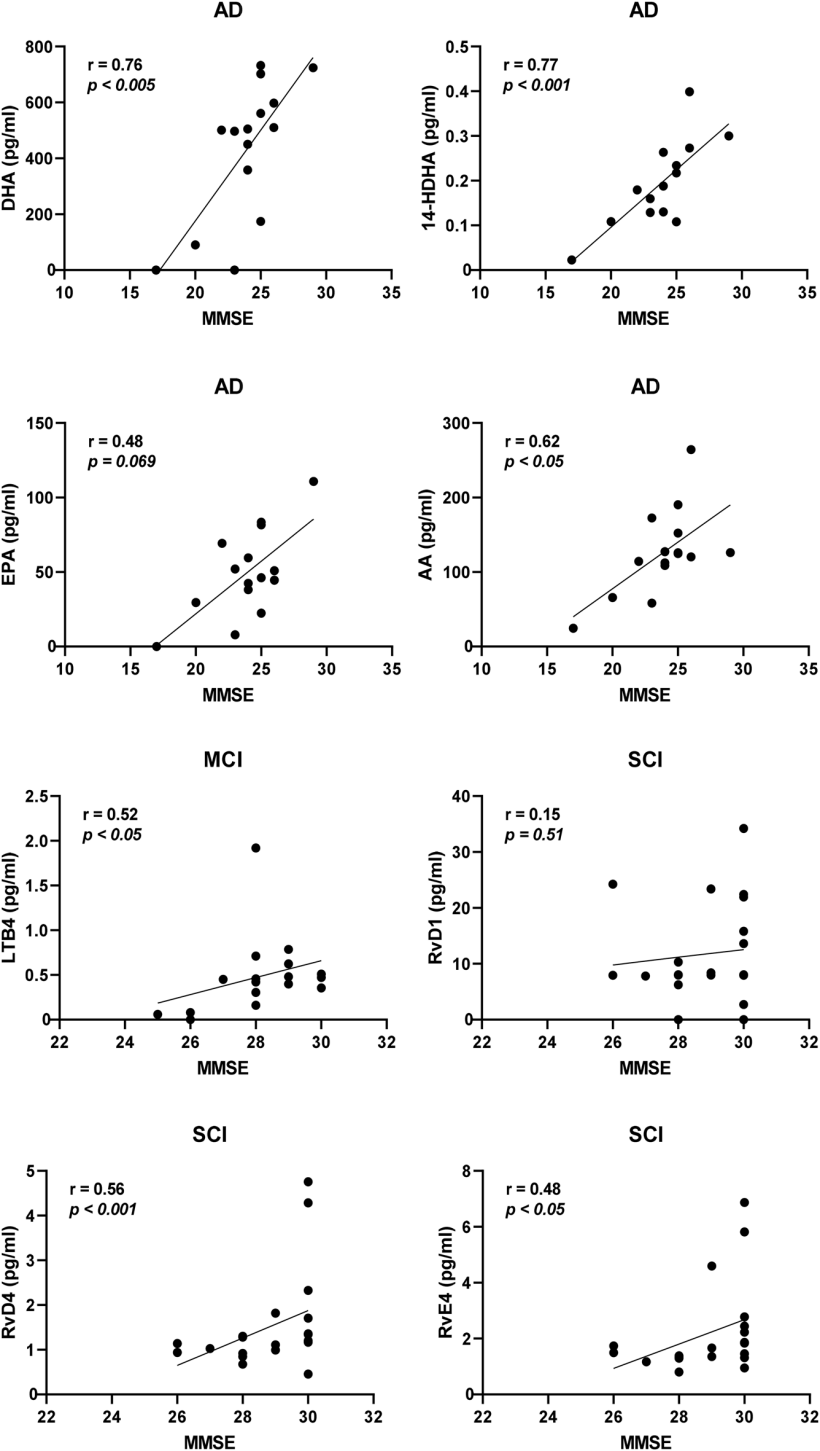
Correlations of bioactive LMs with cognitive function in an age-matched sub-cohort. The levels of lipid mediators (LMs) in cerebrospinal fluid (CSF) samples from the age-matched cohort of patients with Alzheimer’s disease (AD) (*n* = 15), mild cognitive impairment (MCI) (*n* = 17), or subjective cognitive impairment (SCI) (*n* = 21) were correlated to the mini-mental state examination (MMSE) test scores and the r-value according to Spearman rank-order test is given together with the *P*-value. Resolvin (Rv) D4 shows positive correlation to the MMSE scores in the SCI group and when analyzing all three diagnostic groups together (Supplementary Table 4a). Unlike in the entire cohort, the levels of RvD1 did not correlate, but a positive correlation is observed in the MCI group between LTB4 and MMSE scores (*r* = 0.52, *P* < 0.05). The omega-3 fatty acid docosahexaenoic acid (DHA) and the intermediate precursor 14-hydroxy-docosahexaenoic acid (14-HDHA) are positively correlated to the MMSE scores in the AD group, whereas the eicosapentaenoic acid (EPA) is not. The levels of the omega-6 fatty acid arachidonic acid (AA) are also positively correlated to the MMSE scores in the AD group

Separating the cases according to diagnosis provided stronger correlative relationships. In the group of AD cases within the entire cohort, the strongest correlations to MMSE were by DHA and 14-HDHA (*r* = 0.53, *P* < 0.0005 for both; Fig. 4), EPA (*r* = 0.51, *P* < 0.001; Fig. 4), AA (*r* = 0.42, *P* < 0.01; Fig. 4), and 20-HDHA (*r* = 0.33, *P* < 0.05). Among the cases diagnosed with MCI, only one negative correlation was found with MMSE, i.e., MaR1 (*r* = − 0.32, *P* < 0.05). Cases diagnosed with SCI showed positive correlations between MMSE and RvD4 (*r* = 0.42, *P* < 0.005; Fig. 4), RvD1 (*r* = 0.36, *P* < 0.01; Fig. 4), RvE4 (*r* = 0.34, *P* < 0.05), and LTB_4_ (*r* = 0.29, *P* < 0.05). In the group of AD cases within the age-matched cohort, there were positive correlations to MMSE scores for DHA (*r* = 0.76, *P* < 0.005), 14-HDHA (*r* = 0.77, *P* < 0.001), and AA (*r* = 0.62, *P* < 0.05), whereas the correlation to EPA did not reach statistical significance (Fig. 5). Among the cases diagnosed with MCI, there was one positive correlation with MMSE, *i.e.*, for LTB4 (*r* = 0.52, *P* < 0.05; Fig. 5). Similar to the entire cohort, cases diagnosed with SCI in the age-matched cohort showed positive correlations between MMSE and RvD4 (*r* = 0.56, *P* < 0.01; Fig. 5) and RvE4 (*r* = 0.48, *P* < 0.05).

#### Aβ_42_

Analysis of all diagnostic groups together in the entire cohort of cases showed that the CSF levels of Aβ_42_ were positively correlated to the levels of RvD4 (*r* = 0.29, *P* < 0.001), RvE1 (*r* = 0.23, *P* < 0.01), RvD1 (*r* = 0.18, *P* < 0.05), and NPD1 (*r* = 0.18, *P* < 0.05) (Supplementary Table 2b). Also, in the age-matched cohort, there were positive correlations between Aβ42 and RvD4 (*r* = 0.46, *P* < 0.001) and RvE1 (*r* = 0.42, *P* < 0.005) (Supplementary Table 4b).

The analysis of correlations according to diagnostic group within the entire cohort showed a positive correlation between Aβ_42_ and 12-HETE (*r* = 0.42, *P* < 0.01), LXA_4_ (*r* = 0.35, *P* < 0.05), LTB_4_ (*r* = 0.33, *P* < 0.05), and RvE4 (*r* = 0.32, *P* < 0.05) among the AD cases. In cases diagnosed with SCI, there was a positive correlation between Aβ_42_ and RvE1 (*r* = 0.27, *P* < 0.05). In the age-matched cohort, positive correlations were observed in the AD group between Aβ42 and 12-HETE (*r* = 0.58), LTB4 (*r* = 0.52), 14-HDHA (*r* = 0.54), 20-HDHA (*r* = 0.59), and AA (*r* = 0.53), all with a significance level of *P* < 0.05. In the MCI group, there was a negative correlation between Aβ42 and NPD1 (*r* = − 0.52, *P* < 0.05), and in the SCI group, a comparatively strong negative correlation between Aβ42 and MaR2 (*r* = − 0.68, *P* < 0.001).

#### t-Tau and p-Tau

The CSF levels of the tangle biomarkers t-tau and p-tau in the entire cohort showed weak correlative relationships to the lipids analyzed (Supplementary Table 2c, d). Analysis of the entire cohort showed a negative correlation between RvD4 and t-tau (*r* = − 0.17, *P* < 0.05), while there was no correlation to p-tau. Considerably more correlations were found to t-tau in the age-matched cohort (Supplementary Table 4c), i.e., negative correlations to EPA (*r* = − 0.40, *P* < 0.005), DHA (*r* = − 0.33, *P* < 0.05), RvD1 (*r* = − 0.33, *P* < 0.05), MaR1 (*r* = − 0.33, *P* < 0.05), RvE1 (*r* = − 0.27, *P* < 0.05), and 12-HETE (*r* = − 0.32, *P* < 0.05). Similarly, negative correlations were found to p-tau for EPA (r = − 0.38, *P* < 0.005), DHA (*r* = − 0.31, *P* < 0.05), RvD1 (*r* = − 0.32, *P* < 0.05), MaR1 (*r* = − 0.27, *P* < 0.05), and 12-HETE (*r* = − 0.32, *P* < 0.05) (Supplementary Table 4d). A positive correlation was found to LTB4 for both t-tau (*r* = 0.36, *P* < 0.01) and p-tau (*r* = 0.35, *P* < 0.05).

The analysis according to the diagnostic group within the entire cohort showed that for AD cases, MaR1 was negatively correlated to t-tau (*r* = − 0.35, *P* < 0.05), and PGD_2_ was positively correlated to p-tau (*r* = 0.32, *P* < 0.05) (Supplementary Table 2c, d). In cases diagnosed with MCI, there was a negative correlation between the levels t-tau and those of LXA_4_ and 12-HETE (*r* = − 0.33, *P* < 0.05 and *r* = − 0.32, *P* < 0.05, respectively) and between the levels t-tau and LXA_4_ (*r* = − 0.33, *P* < 0.05). There was no correlation to the CSF levels of t- or p-tau within the SCI group. In the age-matched cohort, negative correlations to t-tau were observed in the AD group for DHA (*r* = − 0.71, *P* < 0.005), EPA (*r* = − 0.81, *P* < 0.0005), 17-HDHA (*r* = − 0.56, *P* < 0.05), and 15-HETE (*r* = − 0.58, *P* < 0.05) (Supplementary Table 4c). Negative correlations were also found to p-tau for EPA (*r* = − 0.60, *P* < 0.05) and 15-HETE (*r* = − 0.55, *P* < 0.05) (Supplementary Table 4d). A positive correlation was found between PGD2 and t-tau (*r* = 0.61, *P* < 0.05) and p-tau (*r* = 0.83, *P* = 0.0001). In the SCI group there was a positive correlation between t-tau and RvE4 (*r* = 0.46, *P* = 0.05) and a negative correlation between p-tau and 12-HETE (*r* = − 0.47, *P* = 0.05).

## Discussion

The inflammatory lipidome is dualistic, consisting of pro-inflammatory LMs as well as LMs that end and resolve inflammation while promoting restoration and regeneration of the tissue, i.e., healing (Serhan et al. 2007). We have previously shown decreased levels of pro-resolving LMs in the human AD brain (Lukiw et al. 2005; Wang et al. 2015; Zhu et al. 2016). One of these LMs, LXA_4_, was present in lower levels in the CSF of AD patients compared to those without clinical evidence of memory deficits, i.e., diagnosed with SCI, and both LXA_4_ and RvD1 were positively correlated to the scores from the MMSE test (Wang et al. 2015). In the present study, we have explored how the levels of both pro-inflammatory and pro-resolving LMs are associated with cognitive dysfunction and CSF biomarker levels in a cohort of SCI, MCI, and AD cases. AD cases are often older than MCI and SCI cases, as in this cohort; therefore, we analyzed the resulting data both in the entire cohort of 136 cases and in an age-matched sub-cohort of 53 cases, and the different outcomes are discussed in the following.

**Fig. 6:**
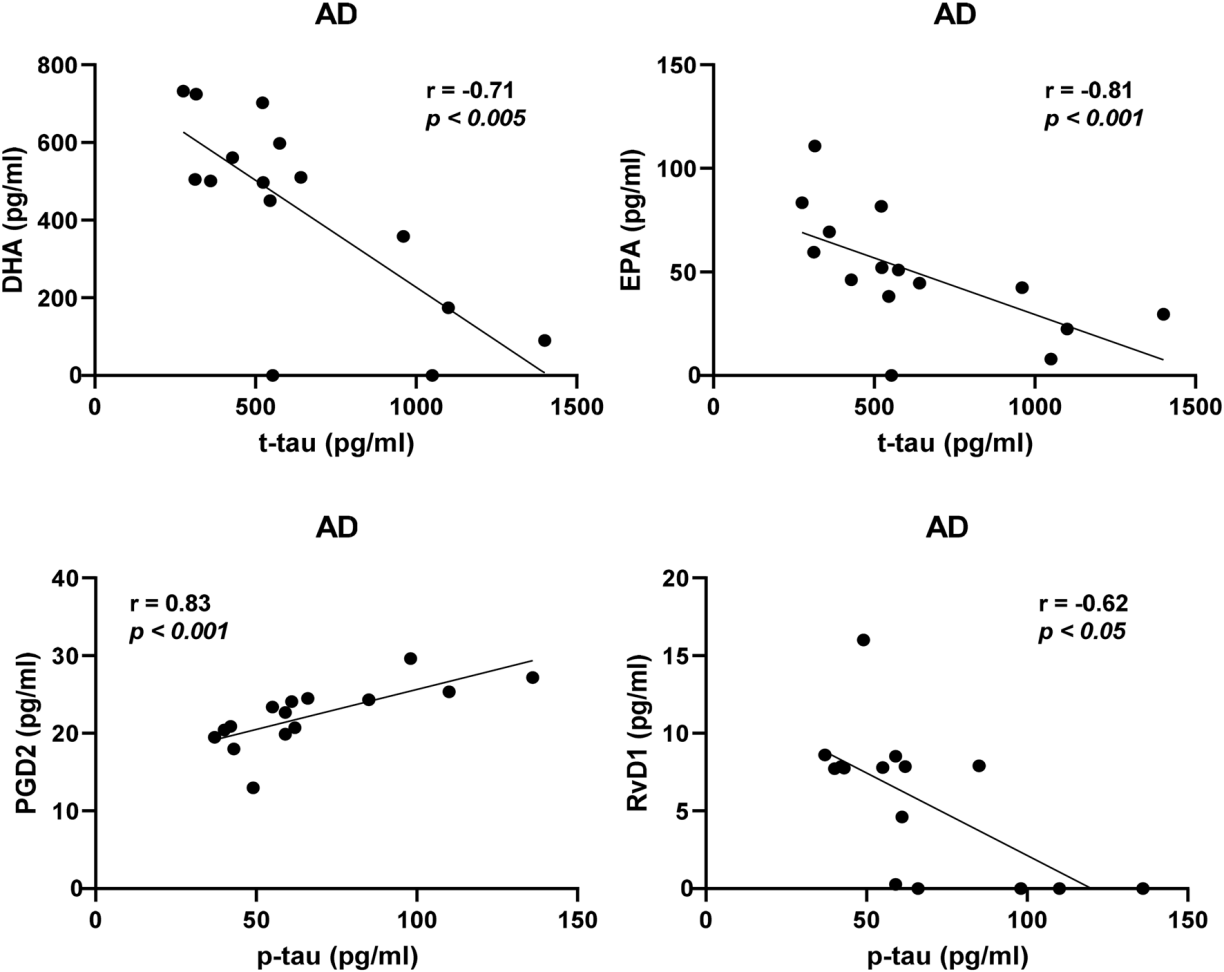
Correlations of bioactive LMs with tau protein in an age-matched sub-cohort. The levels of lipid mediators (LMs) in cerebrospinal fluid (CSF) samples from the age-matched cohort of patients with Alzheimer’s disease (AD) (*n* = 15), mild cognitive impairment (MCI) (*n* = 17), or subjective cognitive impairment (SCI) (*n* = 21) were correlated to the CSF levels of total tau (t-tau) or phosphorylated tau (p-tau) and the r-value according to Spearman rank-order test is given together with the *P*-value (see Supplementary Table 4c, d). In the AD group, resolvin (Rv) D1 shows a negative correlation to the p-tau levels and when analyzing all three diagnostic groups together, whereas prostaglandin (PG) D2 levels correlated positively to the levels of p-tau. The levels of the omega-3 fatty acids docosahexaenoic acid (DHA) and eicosapentaenoic acid (EPA) correlated negatively to t-tau levels

Analysis of the entire cohort showed that the levels of RvD4 and NPD1 were lower in both AD and MCI patients compared to SCI patients, while RvE4 and MaR1 were lower in MCI patients only. Analysis of the age-matched cohort confirmed these findings for RvD4 and RvE4, whereas the differences for NPD1 and MaR1 did not reach statistical significance, indicating that deficiencies in the former two LMs may be more intimately involved in the pathology of AD (Fig. 6).

In the analysis of the entire cohort, differences between the diagnostic groups in RvD4 were also seen in a gender-separated comparison, but this was not evident for NPD1. The difference in MaR1 levels was statistically significant only in men, thus contributing most to the difference seen for all cases. Further analysis within male and female groups showed some additional differences in pro-resolving LMs, such as for RvD3, which was lower in women with MCI than with SCI, for RvE1 that was lower in men with AD than with SCI, and for LXA_4_ that was reduced in men with MCI compared to men with SCI. Moreover, DHA levels in CSF were reduced in men with AD. Both intermediate precursors, 17-HDHA and 15-HETE, were higher in AD, either compared to MCI or to SCI.

Several factors may influence the levels of lipids and give rise to the different results seen in men and women, including diet, age, sex hormones, and the ability to synthesize lipids. Indeed, the ability to synthesize long-chain fatty acids was shown to be higher for women than men, as suggested by a higher conversion rate of α-linoleic acid (ALA) to DHA and EPA (Burdge and Wootton 2002). In a study on mice (Rodriguez-Navas et al. 2016), females had higher brain levels of PUFAs than males, both after a Western-style high fat diet and regular chow diet, while plasma levels were similar.

Although analyses of LTB_4_ in CSF have been performed since the eighties (Westcott et al. 1987), the significance of its presence in CSF in the context of AD is not known. We show that LTB_4_ in CSF of both AD and MCI patients was slightly higher than in SCI and positively correlated to the levels of Aβ_42_ in AD patients. However, the statistical significance was not present in the age-matched cohort, suggesting that it may not be of major importance. However, in studies on multiple sclerosis (MS) (Neu et al. 1992), higher levels of LTB_4_ were found in the CSF of MS patients compared to controls, suggesting LTB_4_ as an indicator of inflammation in the brain. LTB_4_ increased the production of Aβ in neurons in culture (Joshi et al. 2014), providing a direct link to the molecular pathology in AD. In addition, we found that the CSF levels of 15-HETE, an intermediary in the synthesis of LTB_4_, were higher in MCI compared to SCI in both the age-matched cohort and the entire cohort and in women, negatively correlated to MMSE scores. Yao et al. previously detected increased levels of 15-HETE in CSF from AD patients (Yao et al. 2005). Interestingly, the levels of 15-HETE were negatively correlated to both t-tau and p-tau in AD cases within the age-matched cohort.

The pro-inflammatory LMs PGD_2_ and PGE_2_ were lower in the CSF of MCI patients compared to SCI and in the case of PGE_2_, also reduced in AD patients compared to SCI. The reduction in PGE2 levels in MCI compared to SCI was confirmed in the age-matched cohort. Furthermore, a decrease in PGF2a levels was found in both AD and MCI cases within the age-matched cohort. Analysis of human *post-mortem* entorhinal cortex showed higher levels of PGD_2_ in AD compared to non-demented controls (Zhu et al. 2016), and studies on CSF samples showed higher levels of PGE_2_ in patients with probable AD (Montine et al. 1999) and in MCI patients but lower in AD (Combrinck et al. 2006). PGD_2_ synthetase and the PGD_2_ receptor DP1 were upregulated in plaque-associated glia in *post-mortem* AD brains and an AD mouse model (Mohri et al. 2007). PGD_2_ mediated neuronal cell death in in vitro cocultures of neurons and microglia exposed to Aβ_42_ (Bate et al. 2006). PGE_2_ is increased in the CSF of patients with severe MS (Prüss et al. 2013). In a mouse model of AD, PGE_2_ was shown to mediate TNF-α- and presenilin (PS) 1/2-dependent deposition of Aβ (Guan et al. 2019). The literature thus suggests that PGD_2_ and PGE_2_ play harmful roles in AD. The explanation for our findings of lower levels of these factors in the CSF of AD patients will need further studies. However, in addition to the role of PGs at the initiation of an inflammatory response (Serhan and Savill 2005), it is hypothesized that there is a post-resolution immunological activity during which PGE_2_ may exert modulatory and anti-inflammatory effects (Feehan and Gilroy 2019). The reduced levels of PGE_2_ in CSF from AD and MCI patients could thus be seen as a deficit for the post-resolution stage.

Our novel finding of the presence of several bioactive LMs in human CSF highlights their abundance, and the present data on decreased levels of MaR1, NPD1, RvD3 (women), RvD4, RvE1 (men), RvE4, and LXA_4_ (men) in patients with cognitive dysfunction are in line with our previous research showing impaired resolution in AD. The analysis of an age-matched sub-cohort confirmed the reduction, especially of RvD4, i.e., in both AD and MCI, and in addition, the levels of RvD1 were decreased in AD cases within this cohort. The fact that some of the findings in the entire cohort were not observed in the age-matched cohort may be explained by the lower number of cases in the latter sub-cohort. It also emphasizes the importance of correcting for age in light of the high correlation between AD and age and the importance of further investigation in large cohorts where confounding factors are excluded or controlled for. Moreover, the correlation to AD biomarkers, such as t-tau and p-tau, was stronger in the age-matched cohort where a negative correlation was found between RvD1 and p-tau in AD cases, as well as between t-tau and DHA and EPA, respectively, whereas PGD2 levels were positively correlated to p-tau in the AD cases. The presence of this relationship in the AD group, but not in MCI or SCI groups, may be due to less heterogeneity among AD patients who have converged on a pathology that is more or less common and in which MCI and SCI patients have not yet developed, and among which patients may diverge from the pathogenesis of AD.

Of the LMs found to be decreased, NPD1 is the most well-studied, and beneficial effects in the brain have been shown (Lukiw et al. 2005; Bazan 2009; Stark and Bazan 2011), as well as direct protection on human neuronal cells (Zhu et al. 2016). NPD1 here showed a positive correlation to the CSF levels of Aβ_42_, known to be decreased in AD patients. Additionally, NPD1 is protective of photoreceptor cell integrity and function (Mukherjee et al. 2004, 2007; Bazan 2006) and thus relevant to retinal degenerative diseases (Bazan 2007).

The decreased levels of MaR1 in CSF of MCI cases can mainly be attributed to reduced levels in men with MCI. We previously found decreased levels of MaR1 in the hippocampus (Wang et al. 2015) and entorhinal cortex (Zhu et al. 2016) of AD patients and beneficial effects of MaR1 in several cellular models (Zhu et al. 2016; Wang et al. 2021). Surprisingly, there was a negative correlation of MaR1 to the MMSE scores in MCI patients, indicating a more complex nature of immune regulation in this heterogeneous group of patients than previously thought. However, this correlation was not observed in the age-matched cohort.

In general, the correlative relationships between the lipids and cognition and AD CSF biomarkers indicated a positive role, where the levels of AA, DHA, and EPA all showed a comparatively strong positive correlation to cognition in AD cases, while within the group of SCI cases the LMs derived from DHA and EPA showed such a relationship. Also, within the age-matched subgroup, there were positive correlations to cognition in AD cases, including DHA, AA, and 14-HDHA and, in SCI cases, to RvD4 and RvE4.

Of note, we, along with other researchers, consistently detect the presence of pro-resolving LMs in pathological as well as healthy tissues, which adds credibility to an evolving concept of the resolution pathway as an ever-present “care-taker-guardian” of the tissue rather than a response that is elicited only on demand. Studies in animal models of cancer (Sulciner et al. 2018; Panigrahy et al. 2019; Fishbein et al. 2021) provide a fascinating perspective on resolution as a defender of the tissue, adding further support to this concept in which future therapies for disorders that today are hard to treat may be found.

Our results uncovered alterations in the pro-resolving CSF lipidome during the dysfunctions of inflammatory resolution in AD. Our data demonstrate that it is possible to detect bioactive lipids in CSF samples and to show that pro-resolving LMs such as RvD4, RvE4, RvD1, NPD1, MaR1, and RvE1 are reduced in CSF samples from patients with cognitive dysfunction, supporting the disturbance of the resolution of inflammation in the brain. Some of these LMs and the PUFA precursors show positive correlations to MMSE test scores, indicating their relevance for cognitive function. Reducing the confounding effect of age produced a clearer picture of the associations between LMs and data on cognition and CSF AD biomarkers, with stronger and additional correlations, such as those to CSF t-tau and p-tau levels.

### Limitations

This is an explorative study, original in that it uses LC–mass spectrometry to analyze the pro-inflammatory and pro-resolving lipidome in samples from cases of AD and MCI as well as SCI in a reasonably large cohort considering the analysis method. However, further studies on a larger cohort are necessary, especially for increasing the cohort of age-matched cases in light of the importance of age seen when selecting an age-matched subgroup. Although we suggest the use of the CSF lipidomic profile as a biomarker of cognitive decline, its usefulness as a novel biomarker must be determined in replication studies, including longitudinal observations of cognitive decline. We are in the process of performing such studies and hope that the results from the present study motivate other researchers to explore and hopefully confirm the association of alteration in CSF LMs that we believe can be seen in our data. Age and gender were included in our analyses, and the influence of gender on the abundance of LMs is also of importance and requires further investigation.

The majority of the differences observed reach the threshold of *P*-values 0.005 or 0.001 and in some cases even 0.0001. Considering the explorative nature and novel findings in our study, the analyses resulting in a *P*-value of > 0.005 should be interpreted with caution and as an impetus for further investigation rather than hard evidence. Although we did not perform a sensitivity power analysis prior to our investigation, we believe that the sample size in our cohort is large enough for an explorative study. Therefore, the fact that MaR1 and RvE4 were decreased only in MCI compared to SCI, whereas NPD1 and RvD4 were reduced in both AD and MCI, may be a reflection of a limited sample size. The findings from analyzing an age-matched subgroup further support this notion.

## Supplementary Information

Below is the link to the electronic supplementary material.

Supplementary file1 (DOCX 356 KB)

Supplementary file2 (DOCX 71 KB)

## References

[CR1] Albert MS, DeKosky ST, Dickson D et al (2011) The diagnosis of mild cognitive impairment due to Alzheimer’s disease: recommendations from the National Institute on Aging-Alzheimer’s Association workgroups on diagnostic guidelines for Alzheimer’s disease. Alzheimers Dement 7:270–279. 10.1016/j.jalz.2011.03.008 21514249 10.1016/j.jalz.2011.03.008 PMC3312027

[CR2] Alzheimer’s Association (2021) 2021 Alzheimer’s disease facts and figures. Alzheimers Dement 17:327–406. 10.1002/alz.12328 33756057 10.1002/alz.12328

[CR3] Arevalo-Rodriguez I, Smailagic N, Roqué I, Figuls M et al (2015) Mini-mental state examination (MMSE) for the detection of Alzheimer’s disease and other dementias in people with mild cognitive impairment (MCI). Cochrane Database Syst Rev. 10.1002/14651858.CD010783.pub2 25740785 10.1002/14651858.CD010783.pub2 PMC6464748

[CR4] Bate C, Kempster S, Williams A (2006) Prostaglandin D2 mediates neuronal damage by amyloid-beta or prions which activates microglial cells. Neuropharmacology 50:229–237. 10.1016/j.neuropharm.2005.09.008 16289250 10.1016/j.neuropharm.2005.09.008

[CR56] Bazan NG (2006) Cell survival matters: Docosahexaenoic acid signaling neuroprotection and photoreceptors. Trends Neurosci 29(5):263–271. 10.1016/j.tins.2006.03.005 16580739 10.1016/j.tins.2006.03.005

[CR57] Bazan NG (2007) Homeostatic regulation of photoreceptor cell integrity: Significance of the potent mediator neuroprotectin D1 biosynthesized from docosahexaenoic acid: The proctor lecture. Investig Ophthalmol Vis Sci 48(11):4866. 10.1167/iovs.07-0918 17962433 10.1167/iovs.07-0918

[CR5] Bazan NG (2009) Cellular and molecular events mediated by docosahexaenoic acid-derived neuroprotectin D1 signaling in photoreceptor cell survival and brain protection. Prostaglandins Leukot Essent Fatty Acids 81:205–211. 10.1016/j.plefa.2009.05.024 19520558 10.1016/j.plefa.2009.05.024 PMC2756692

[CR6] Buckley CD, Gilroy DW, Serhan CN (2014) Proresolving lipid mediators and mechanisms in the resolution of acute inflammation. Immunity 40:315–327. 10.1016/j.immuni.2014.02.009 24656045 10.1016/j.immuni.2014.02.009 PMC4004957

[CR7] Burdge GC, Wootton SA (2002) Conversion of alpha-linolenic acid to eicosapentaenoic, docosapentaenoic and docosahexaenoic acids in young women. Br J Nutr 88:411–420. 10.1079/BJN2002689 12323090 10.1079/BJN2002689

[CR8] Coceani F, Bishai I, Lees J, Sirko S (1986) Prostaglandin E2 and fever: a continuing debate. Yale J Biol Med 59:169–174 3488620 PMC2590134

[CR9] Combrinck M, Williams J, De Berardinis MA et al (2006) Levels of CSF prostaglandin E2, cognitive decline, and survival in Alzheimer’s disease. J Neurol Neurosurg Psychiatry 77:85–88. 10.1136/jnnp.2005.063131 15944180 10.1136/jnnp.2005.063131 PMC2117387

[CR10] Dunn HC, Ager RR, Baglietto-Vargas D et al (2015) Restoration of lipoxin A4 signaling reduces Alzheimer’s disease-like pathology in the 3xTg-AD mouse model. J Alzheimers Dis 43:893–903. 10.3233/JAD-141335 25125468 10.3233/JAD-141335 PMC4319708

[CR11] Emre C, Hjorth E, Bharani K et al (2020) Receptors for pro-resolving mediators are increased in Alzheimer’s disease brain. Brain Pathol 30:614–640. 10.1111/bpa.12812 31912564 10.1111/bpa.12812 PMC8018009

[CR12] Emre C, Arroyo-García LE, Do KV et al (2022) Intranasal delivery of pro-resolving lipid mediators rescue memory and gamma oscillation impairment in AppNL-G-F/NL-G-F mice. Commun Biol. 10.1038/s42003-022-03169-3 35314851 10.1038/s42003-022-03169-3 PMC8938447

[CR13] Feehan KT, Gilroy DW (2019) Is resolution the end of inflammation? Trends Mol Med 25:198–214. 10.1016/j.molmed.2019.01.006 30795972 10.1016/j.molmed.2019.01.006

[CR14] Fishbein A, Hammock BD, Serhan CN, Panigrahy D (2021) Carcinogenesis: failure of resolution of inflammation? Pharmacol Ther 218:107670. 10.1016/j.pharmthera.2020.107670 32891711 10.1016/j.pharmthera.2020.107670 PMC7470770

[CR15] Folch J, Lees M, Sloane Stanley GH (1957) A simple method for the isolation and purification of total lipides from animal tissues. J Biol Chem 226:497–509 13428781

[CR16] Folstein MF, Folstein SE, McHugh PR (1975) “Mini-mental state”. A practical method for grading the cognitive state of patients for the clinician. J Psychiatr Res 12:189–198. 10.1016/0022-3956(75)90026-6 1202204 10.1016/0022-3956(75)90026-6

[CR17] Gonzalez-Gay MA, Gonzalez-Juanatey C, Llorca J et al (2008) Contribution of HLA-DRB1 shared epitope alleles and chronic inflammation to the increased incidence of cardiovascular disease in rheumatoid arthritis: comment on the article by Farragher et al. Arthritis Rheum 58:2584. 10.1002/art.23637 18668593 10.1002/art.23637

[CR18] Guan P-P, Liang Y-Y, Cao L-L et al (2019) Cyclooxygenase-2 induced the β-amyloid protein deposition and neuronal apoptosis via upregulating the synthesis of prostaglandin E2 and 15-deoxy-δ12,14-prostaglandin J2. Neurotherapeutics 16:1255–1268. 10.1007/s13311-019-00770-z 31392591 10.1007/s13311-019-00770-z PMC6985346

[CR19] Heneka MT, Carson MJ, El Khoury J et al (2015) Neuroinflammation in Alzheimer’s disease. Lancet Neurol 14:388–405. 10.1016/S1474-4422(15)70016-5 25792098 10.1016/S1474-4422(15)70016-5 PMC5909703

[CR20] Jack CR, Bennett DA, Blennow K et al (2018) NIA-AA research framework: toward a biological definition of Alzheimer’s disease. Alzheimers Dement 14:535–562. 10.1016/j.jalz.2018.02.018 29653606 10.1016/j.jalz.2018.02.018 PMC5958625

[CR21] Joshi YB, Di Meco A, Praticó D (2014) Modulation of amyloid-β production by leukotriene B4 via the γ-secretase pathway. J Alzheimers Dis 38:503–506. 10.3233/JAD-131223 24008686 10.3233/JAD-131223

[CR22] Juan H (1978) Prostaglandins as modulators of pain. Gen Pharmacol 9:403–409. 10.1016/0306-3623(78)90025-3 365674 10.1016/0306-3623(78)90025-3

[CR23] Kantarci A, Aytan N, Palaska I et al (2018) Combined administration of resolvin E1 and lipoxin A4 resolves inflammation in a murine model of Alzheimer’s disease. Exp Neurol 300:111–120. 10.1016/j.expneurol.2017.11.005 29126887 10.1016/j.expneurol.2017.11.005

[CR24] Kozak W, Fraifeld V (2004) Non-prostaglandin eicosanoids in fever and anapyrexia. Front Biosci 9:3339–3355. 10.2741/1486 15353362 10.2741/1486

[CR25] Lee JY, Han SH, Park MH et al (2020) N-AS-triggered SPMs are direct regulators of microglia in a model of Alzheimer’s disease. Nat Commun 11:2358. 10.1038/s41467-020-16080-4 32398649 10.1038/s41467-020-16080-4 PMC7217877

[CR26] Lue LF, Brachova L, Civin WH, Rogers J (1996) Inflammation, a beta deposition, and neurofibrillary tangle formation as correlates of Alzheimer’s disease neurodegeneration. J Neuropathol Exp Neurol 55:1083–1088 8858005

[CR27] Luis CA, Keegan AP, Mullan M (2009) Cross validation of the Montreal cognitive assessment in community dwelling older adults residing in the Southeastern US. Int J Geriatr Psychiatry 24:197–201. 10.1002/gps.2101 18850670 10.1002/gps.2101

[CR28] Lukiw WJ, Cui J-G, Marcheselli VL et al (2005) A role for docosahexaenoic acid-derived neuroprotectin D1 in neural cell survival and Alzheimer disease. J Clin Invest 115:2774–2783. 10.1172/JCI25420 16151530 10.1172/JCI25420 PMC1199531

[CR29] Maccioni RB, Muñoz JP, Barbeito L (2001) The molecular bases of Alzheimer’s disease and other neurodegenerative disorders. Arch Med Res 32:367–381. 10.1016/s0188-4409(01)00316-2 11578751 10.1016/s0188-4409(01)00316-2

[CR30] McGeer PL, McGeer EG (1995) The inflammatory response system of brain: implications for therapy of Alzheimer and other neurodegenerative diseases. Brain Res Brain Res Rev 21:195–218. 10.1016/0165-0173(95)00011-9 8866675 10.1016/0165-0173(95)00011-9

[CR31] Medeiros R, Kitazawa M, Passos GF et al (2013) Aspirin-triggered lipoxin A4 stimulates alternative activation of microglia and reduces Alzheimer disease-like pathology in mice. Am J Pathol 182:1780–1789. 10.1016/j.ajpath.2013.01.051 23506847 10.1016/j.ajpath.2013.01.051 PMC3644736

[CR32] Mohri I, Kadoyama K, Kanekiyo T et al (2007) Hematopoietic prostaglandin D synthase and DP1 receptor are selectively upregulated in microglia and astrocytes within senile plaques from human patients and in a mouse model of Alzheimer disease. J Neuropathol Exp Neurol 66:469–480. 10.1097/01.jnen.0000240472.43038.27 17549007 10.1097/01.jnen.0000240472.43038.27

[CR33] Montine TJ, Sidell KR, Crews BC et al (1999) Elevated CSF prostaglandin E2 levels in patients with probable AD. Neurology 53:1495–1498. 10.1212/wnl.53.7.1495 10534257 10.1212/wnl.53.7.1495

[CR58] Mukherjee PK, Marcheselli VL, Barreiro S et al (2007) Neurotrophins enhance retinal pigment epithelial cell survival through neuroprotectin D1 signaling.Proc Natl Acad Sci 104(32):13152–13157. 10.1073/pnas.0705949104 17670936 10.1073/pnas.0705949104 PMC1941803

[CR59] Mukherjee PK, Marcheselli VL, Serhan CN et al (2004) Neuroprotectin D1: A docosahexaenoic acid-derived docosatriene protects human retinal pigment epithelial cells from oxidative stress.Proc Natl Acad Sci 101(32):8491–8496. 10.1073/pnas.0402531101 15152078 10.1073/pnas.0402531101 PMC420421

[CR34] Naik M, Nygaard HA (2008) Diagnosing dementia—ICD-10 not so bad after all: a comparison between dementia criteria according to DSM-IV and ICD-10. Int J Geriatr Psychiatry 23:279–282. 10.1002/gps.1874 17702050 10.1002/gps.1874

[CR35] Neu I, Mallinger J, Wildfeuer A, Mehlber L (1992) Leukotrienes in the cerebrospinal fluid of multiple sclerosis patients. Acta Neurol Scand 86:586–587. 10.1111/j.1600-0404.1992.tb05491.x 1336293 10.1111/j.1600-0404.1992.tb05491.x

[CR36] Olsson B, Lautner R, Andreasson U et al (2016) CSF and blood biomarkers for the diagnosis of Alzheimer’s disease: a systematic review and meta-analysis. Lancet Neurol 15:673–684. 10.1016/S1474-4422(16)00070-3 27068280 10.1016/S1474-4422(16)00070-3

[CR37] Panigrahy D, Gartung A, Yang J et al (2019) Preoperative stimulation of resolution and inflammation blockade eradicates micrometastases. J Clin Invest 129:2964–2979. 10.1172/JCI127282 31205032 10.1172/JCI127282 PMC6597207

[CR38] Prince M, Bryce R, Albanese E et al (2013) The global prevalence of dementia: a systematic review and metaanalysis. Alzheimers Dement 9:63–75. 10.1016/j.jalz.2012.11.007 23305823 10.1016/j.jalz.2012.11.007

[CR39] Prüss H, Rosche B, Sullivan AB et al (2013) Proresolution lipid mediators in multiple sclerosis—differential, disease severity-dependent synthesis—a clinical pilot trial. PLoS ONE 8:e55859. 10.1371/journal.pone.0055859 23409068 10.1371/journal.pone.0055859 PMC3568070

[CR40] Reisberg B, Prichep L, Mosconi L et al (2008) The pre-mild cognitive impairment, subjective cognitive impairment stage of Alzheimer’s disease. Alzheimers Dement 4:S98–S108. 10.1016/j.jalz.2007.11.017 18632010 10.1016/j.jalz.2007.11.017

[CR41] Rodriguez-Navas C, Morselli E, Clegg DJ (2016) Sexually dimorphic brain fatty acid composition in low and high fat diet-fed mice. Mol Metab 5:680–689. 10.1016/j.molmet.2016.06.014 27656405 10.1016/j.molmet.2016.06.014 PMC5021676

[CR42] Rodriguez-Vieitez E, Saint-Aubert L, Carter SF et al (2016) Diverging longitudinal changes in astrocytosis and amyloid PET in autosomal dominant Alzheimer’s disease. Brain 139:922–936. 10.1093/brain/awv404 26813969 10.1093/brain/awv404 PMC4766380

[CR43] Scheltens P, Blennow K, Breteler MMB et al (2016) Alzheimer’s disease. Lancet 388:505–517. 10.1016/S0140-6736(15)01124-1 26921134 10.1016/S0140-6736(15)01124-1

[CR44] Serhan CN, Savill J (2005) Resolution of inflammation: the beginning programs the end. Nat Immunol 6:1191–1197. 10.1038/ni1276 16369558 10.1038/ni1276

[CR45] Serhan CN, Brain SD, Buckley CD et al (2007) Resolution of inflammation: state of the art, definitions and terms. FASEB J 21:325–332. 10.1096/fj.06-7227rev 17267386 10.1096/fj.06-7227rev PMC3119634

[CR46] Serhan CN, Chiang N, Dalli J, Levy BD (2014) Lipid mediators in the resolution of inflammation. Cold Spring Harb Perspect Biol 7:a016311. 10.1101/cshperspect.a016311 25359497 10.1101/cshperspect.a016311 PMC4315926

[CR47] Stark DT, Bazan NG (2011) Neuroprotectin D1 induces neuronal survival and downregulation of amyloidogenic processing in Alzheimer’s disease cellular models. Mol Neurobiol 43:131–138. 10.1007/s12035-011-8174-4 21431475 10.1007/s12035-011-8174-4 PMC12866416

[CR48] Sulciner ML, Serhan CN, Gilligan MM et al (2018) Resolvins suppress tumor growth and enhance cancer therapy. J Exp Med 215:115–140. 10.1084/jem.20170681 29191914 10.1084/jem.20170681 PMC5748851

[CR49] Wang X, Zhu M, Hjorth E et al (2015) Resolution of inflammation is altered in Alzheimer’s disease. Alzheimers Dement 11(40–50):e1–e2. 10.1016/j.jalz.2013.12.024 24530025 10.1016/j.jalz.2013.12.024 PMC4275415

[CR50] Wang Y, Leppert A, Tan S et al (2021) Maresin 1 attenuates pro-inflammatory activation induced by β-amyloid and stimulates its uptake. J Cell Mol Med 25:434–447. 10.1111/jcmm.16098 33225628 10.1111/jcmm.16098 PMC7810927

[CR51] Westcott JY, Murphy RC, Stenmark K (1987) Eicosanoids in human ventricular cerebrospinal fluid following severe brain injury. Prostaglandins 34:877–887. 10.1016/0090-6980(87)90068-2 2835791 10.1016/0090-6980(87)90068-2

[CR52] Winblad B, Palmer K, Kivipelto M et al (2004) Mild cognitive impairment–beyond controversies, towards a consensus: report of the International Working Group on Mild Cognitive Impairment. J Intern Med 256:240–246. 10.1111/j.1365-2796.2004.01380.x 15324367 10.1111/j.1365-2796.2004.01380.x

[CR53] Yao Y, Clark CM, Trojanowski JQ et al (2005) Elevation of 12/15 lipoxygenase products in AD and mild cognitive impairment. Ann Neurol 58:623–626. 10.1002/ana.20558 16037976 10.1002/ana.20558

[CR54] Yin P, Wang X, Wang S et al (2019) Maresin 1 improves cognitive decline and ameliorates inflammation in a mouse model of Alzheimer’s disease. Front Cell Neurosci 13:466. 10.3389/fncel.2019.00466 31680874 10.3389/fncel.2019.00466 PMC6803487

[CR55] Zhu M, Wang X, Hjorth E et al (2016) Pro-resolving lipid mediators improve neuronal survival and increase Aβ42 phagocytosis. Mol Neurobiol 53:2733–2749. 10.1007/s12035-015-9544-0 26650044 10.1007/s12035-015-9544-0 PMC4824659

