# Supplementary material for: Cerebrospinal Fluid Profile of Lipid Mediators in Alzheimer’s Disease
Source: Cell Mol Neurobiol. 2022 Apr 1;43(2):797–811. doi: 10.1007/s10571-022-01216-5 (PMC9957874; doi:10.1007/s10571-022-01216-5)
Supplement: Supplementary file 1 — Supplementary file1 (DOCX 356 KB) [file 10571_2022_1216_MOESM1_ESM.docx]

**Cerebrospinal fluid profile of lipid mediators in Alzheimer’s disease**

Cellular and Molecular Neurobiology

Khanh V. Do, Erik Hjorth, Ying Wang, Bokkyoo Jun, Marie-Audrey I. Kautzmann, Makiko Ohshima, Maria Eriksdotter, Marianne Schultzberg, Nicolas G. Bazan

*Corresponding Authors: Marianne Schultzberg and Nicolas G. Bazan

E-mail: [Marianne.Schultzberg@ki.se](mailto:Marianne.Schultzberg@ki.se) and [NBazan@lsuhsc.edu](mailto:NBazan@lsuhsc.edu)

**Supplementary Fig. 1. Metabolic pathways of lipid mediators.** Pro-resolving and pro-inflammatory lipid mediators (LMs) are derived from the omega-3 and -6 polyunsaturated fatty acids (PUFAs) docosahexaenoic acid (DHA), eicosapentaenoic acid (EPA), and arachidonic acid (AA). The figure shows the LMs analyzed in the present study and the biosynthetic enzymes involved in their synthesis. COX = cyclooxygenase, GPX = glutathione peroxidase, HDHA = hydroxydocosahexaenoic acid, HEPE = hydroxyeicosapentaenoic acid, HETE = hydroxyeicosatetraenoic acid, LOX = lipoxygenase, LT = leukotriene, LX = lipoxin, MaR = maresin, NPD = neuroprotectin D1, PG = prostaglandin, Rv = resolvin.

**Supplementary Fig. 2. Analysis of differences in LMs between diagnostic groups within each gender separately.** LMs were assessed in the cerebrospinal fluid (CSF) samples from patients with Alzheimer's disease (AD) (F = 24, M = 16), mild cognitive impairment (MCI) (F = 23, M = 20), or subjective cognitive impairment (SCI) (F = 33, M = 20), using liquid chromatography-tandem mass spectrometry (LC-MS/MS). Comparisons were performed between diagnostic groups within the male and female cases, respectively. The analyses show differences that were not present when analyzing data from men and women together (see Fig. 1), such as for resolvin (Rv) E1, lipoxin (LX) A4, RvD3, and docosahexaenoic acid (DHA). The analysis also indicates which gender is important for showing the differences when analyzing data from men and women together, such as for maresin 1 (MaR1). Comparisons between groups were performed by Kruskal-Wallis ANOVA with Dunn’s multiple comparisons post hoc test, (*P < 0.05, **P < 0.005, ***P < 0.001, ****P < 0.0001).
