# Supplementary material for: Cerebrospinal Fluid Profile of Lipid Mediators in Alzheimer’s Disease
Source: Cell Mol Neurobiol. 2022 Apr 1;43(2):797–811. doi: 10.1007/s10571-022-01216-5 (PMC9957874; doi:10.1007/s10571-022-01216-5)
Supplement: Supplementary file 2 — Supplementary file2 (DOCX 71 KB) [file 10571_2022_1216_MOESM2_ESM.docx]

**Cerebrospinal fluid profile of lipid mediators in Alzheimer’s disease**

Cellular and Molecular Neurobiology

Khanh V. Do, Erik Hjorth, Ying Wang, Bokkyoo Jun, Marie-Audrey I. Kautzmann, Makiko Ohshima, Maria Eriksdotter, Marianne Schultzberg, Nicolas G. Bazan

*Corresponding Authors: Marianne Schultzberg and Nicolas G. Bazan

E-mail: [Marianne.Schultzberg@ki.se](mailto:Marianne.Schultzberg@ki.se) and [NBazan@lsuhsc.edu](mailto:NBazan@lsuhsc.edu)

**Supplementary Table 1. LMs in CSF from patients diagnosed with AD, MCI, or SCI within the entire cohort.**

| LM | AD | | | MCI | | | SCI | | |
| --- | --- | --- | --- | --- | --- | --- | --- | --- | --- |
|  | Median | Lower Q | Upper Q | Median | Lower Q | Upper Q | Median | Lower Q | Upper Q |
| RvD1 | 7.85 | *3.72* | *8.06* | 7.98 | *7.72* | *8.09* | 7.95 | *7.82* | *8.38* |
| RvD3 | 0.76 | *0.61* | *0.91* | 0.69 | *0.58* | *0.90* | 0.78 | *0.67* | *1.17* |
| RvD4 | 0.93 | *0.78* | *1.01* | 0.94 | *0.87* | *1,00* | 1.10 | *0.94* | *1.35* |
| MaR1 | 0.24 | *0.16* | *0.34* | 0.19 | *0.14* | *0.24* | 0.26 | *0.18* | *0.33* |
| MaR2 | 0.20 | *0.17* | *0.26* | 0.20 | *0.15* | *0.23* | 0.20 | *0.18* | *0.26* |
| NPD1 | 0.30 | *0.25* | *0.37* | 0.30 | *0.22* | *0.36* | 0.38 | *0.29* | *0.50* |
| RvE1 | 0.95 | *0.73* | *1.27* | 0.93 | *0.78* | *1.35* | 1.18 | *0.93* | *1.53* |
| RvE4 | 1.36 | *0.94* | *1.54* | 1.23 | *1.06* | *1.46* | 1.39 | *1.28* | *1.74* |
| LXA4 | 0.42 | *0.35* | *0.56* | 0.43 | *0.37* | *0.51* | 0.48 | *0.41* | *0.64* |
| LTB4 | 0.45 | *0.32* | *0.64* | 0.42 | *0.23* | *0.62* | 0.32 | *0.20* | *0.40* |
| 14-HDHA | 0.20 | *0.14* | *0.25* | 0.19 | *0.15* | *0.23* | 0.17 | *0.13* | *0.24* |
| 17-HDHA | 0.22 | *0.17* | *0.31* | 0.17 | *0.13* | *0.23* | 0.20 | *0.14* | *0.25* |
| 20-HDHA | 0.76 | *0.61* | *0.96* | 0.83 | *0.59* | *1.00* | 0.67 | *0.54* | *1.01* |
| 12-HETE | 1.32 | *0.28* | *1.76* | 1.22 | *1.01* | *1.66* | 1.08 | *0.00* | *1.55* |
| 15-HETE | 0.99 | *0.43* | *1.22* | 0.84 | *0.45* | *1.48* | 0.40 | *0.20* | *0.74* |
| PGD2 | 22.06 | *20.37* | *23.91* | 20.53 | *19.49* | *22.47* | 29.74 | *20.46* | *38.53* |
| PGE2 | 28.86 | *25.04* | *32.10* | 28.39 | *26.16* | *30.08* | 35.43 | *27.56* | *57.11* |
| PGF2a | 10.50 | *4.95* | *11.73* | 10.28 | *9.04* | *11.02* | 9.35 | *8.04* | *13.34* |
| DHA | 503.75 | *381.83* | *582.30* | 539.71 | *457.56* | *713.61* | 498.17 | *421.98* | *702.67* |
| EPA | 54.90 | *38.05* | *84.34* | 67.23 | *45.06* | *85.02* | 58.65 | *42.92* | *84.08* |
| AA | 138.57 | *112.77* | *161.06* | 143.13 | *103.19* | *187.31* | 116.21 | *95.79* | *186.70* |

The levels of lipid mediators (LMs) and polyunsaturated fatty acids (PUFAs) in cerebrospinal fluid (CSF) samples of the entire cohort of individuals with Alzheimer's disease (AD), mild cognitive impairment (MCI) and subjective cognitive impairment (SCI) are presented as median and interquartile (Q) range in fg/ml. AA = arachidonic acid, DHA = docosahexaenoic acid, EPA = eicosapentaenoic acid, HDHA = hydroxy-docosahexaenoic acids, HETE = hydroxy-eicosatetraenoic, LT = leukotriene, LX = lipoxin, MaR = maresin, NPD = neuroprotectin D, PG = prostaglandin, Rv = resolvin

**Supplementary Table 2a-d. Correlation of LMs to cognition and CSF plaque and tangle biomarkers (entire cohort).**

| MMSE | AD | | MCI | | SCI | | All | |
| --- | --- | --- | --- | --- | --- | --- | --- | --- |
|  | r | *P* value | r | *P* value | r | *P* value | r | *P* value |
| RvD1 | -0.24 | n.s. | -0.12 | n.s. | **0.36** | **0.0090** | **0.17** | **0.0434** |
| RvD3 | -0.05 | n.s. | 0.12 | n.s. | 0.14 | n.s. | 0.17 | n.s. |
| RvD4 | -0.13 | n.s. | -0.22 | n.s. | **0.42** | **0.0018** | **0.29** | **0.0007** |
| MaR1 | 0.09 | n.s. | **-0.32** | **0.0377** | 0.14 | n.s. | 0.02 | n.s. |
| MaR2 | -0.12 | n.s. | -0.27 | n.s. | 0.12 | n.s. | -0.01 | n.s. |
| NPD1 | -0.25 | n.s. | 0.11 | n.s. | -0.22 | n.s. | 0.05 | n.s. |
| RvE1 | 0.03 | n.s. | -0.15 | n.s. | 0.16 | n.s. | 0.16 | n.s. |
| RvE4 | -0.03 | n.s. | -0.21 | n.s. | **0.34** | **0.0125** | 0.11 | n.s. |
| LXA4 | -0.13 | n.s. | -0.12 | n.s. | 0.07 | n.s. | 0.07 | n.s. |
| LTB4 | 0.02 | n.s. | 0.19 | n.s. | **0.29** | **0.0351** | -0.09 | n.s. |
| 14-HDHA | **0.53** | **0.0004** | 0.19 | n.s. | 0.18 | n.s. | 0.13 | n.s. |
| 17-HDHA | 0.18 | n.s. | 0.02 | n.s. | 0.14 | n.s. | -0.09 | n.s. |
| 20-HDHA | **0.33** | **0.0355** | 0.30 | n.s. | -0.06 | n.s. | 0.04 | n.s. |
| 12-HETE | 0.02 | n.s. | -0.06 | n.s. | -0.02 | n.s. | -0.08 | n.s. |
| 15-HETE | 0.04 | n.s. | -0.12 | n.s. | 0.12 | n.s. | -0.15 | n.s. |
| PGD2 | -0.03 | n.s. | -0.02 | n.s. | 0.01 | n.s. | 0.11 | n.s. |
| PGE2 | 0.02 | n.s. | -0.10 | n.s. | 0.01 | n.s. | **0.18** | **0.0395** |
| PGF2a | -0.04 | n.s. | -0.27 | n.s. | 0.12 | n.s. | 0.01 | n.s. |
| DHA | **0.53** | **0.0005** | 0.09 | n.s. | 0.10 | n.s. | **0.21** | **0.0143** |
| EPA | **0.51** | **0.0008** | 0.05 | n.s. | 0.16 | n.s. | **0.18** | **0.0373** |
| AA | **0.42** | **0.0066** | 0.04 | n.s. | 0.01 | n.s. | 0.04 | n.s. |

| Aβ_42_ | AD | | MCI | | SCI | | All | |
| --- | --- | --- | --- | --- | --- | --- | --- | --- |
|  | r | P value | r | P value | r | P value | r | P value |
| RvD1 | 0.14 | n.s. | 0.23 | n.s. | -0.14 | n.s. | **0.18** | **0.0319** |
| RvD3 | 0.14 | n.s. | 0.23 | n.s. | -0.09 | n.s. | 0.11 | n.s. |
| RvD4 | 0.15 | n.s. | 0.14 | n.s. | 0.11 | n.s. | **0.29** | **0.0007** |
| MaR1 | 0.23 | n.s. | 0.19 | n.s. | 0.13 | n.s. | 0.10 | n.s. |
| MaR2 | 0.30 | n.s. | -0.19 | n.s. | -0.26 | n.s. | -0.07 | n.s. |
| NPD1 | -0.09 | n.s. | 0.03 | n.s. | 0.12 | n.s. | **0.18** | **0.0398** |
| RvE1 | -0.08 | n.s. | 0.19 | n.s. | **0.27** | **0.0474** | **0.23** | **0.0060** |
| RvE4 | **0.32** | **0.0437** | 0.03 | n.s. | -0.07 | n.s. | 0.10 | n.s. |
| LXA4 | **0.35** | **0.0279** | -0.08 | n.s. | 0.05 | n.s. | 0.15 | n.s. |
| LTB4 | **0.33** | **0.0375** | -0.05 | n.s. | 0.00 | n.s. | -0.15 | n.s. |
| 14-HDHA | 0.02 | n.s. | 0.09 | n.s. | -0.20 | n.s. | -0.18 | n.s. |
| 17-HDHA | 0.22 | n.s. | -0.10 | n.s. | -0.06 | n.s. | -0.17 | n.s. |
| 20-HDHA | 0.12 | n.s. | 0.04 | n.s. | -0.17 | n.s. | -0.06 | n.s. |
| 12-HETE | **0.42** | **0.0064** | -0.05 | n.s. | -0.08 | n.s. | -0.05 | n.s. |
| 15-HETE | 0.29 | n.s. | -0.04 | n.s. | -0.11 | n.s. | -0.14 | n.s. |
| PGD2 | -0.25 | n.s. | -0.14 | n.s. | 0.00 | n.s. | -0.01 | n.s. |
| PGE2 | -0.14 | n.s. | -0.24 | n.s. | 0.00 | n.s. | 0.11 | n.s. |
| PGF2a | **0.32** | **0.0440** | -0.05 | n.s. | -0.08 | n.s. | 0.01 | n.s. |
| DHA | 0.09 | n.s. | -0.15 | n.s. | -0.09 | n.s. | 0.04 | n.s. |
| EPA | 0.15 | n.s. | -0.01 | n.s. | -0.07 | n.s. | 0.04 | n.s. |
| AA | 0.22 | n.s. | -0.12 | n.s. | -0.08 | n.s. | -0.05 | n.s. |

| t-tau | AD | | MCI | | SCI | | All | |
| --- | --- | --- | --- | --- | --- | --- | --- | --- |
|  | r | P value | r | P value | r | P value | r | P value |
| RvD1 | -0.16 | n.s. | -0.05 | n.s. | 0.03 | n.s. | -0.16 | n.s. |
| RvD3 | 0.00 | n.s. | -0.24 | n.s. | -0.05 | n.s. | -0.13 | n.s. |
| RvD4 | -0.03 | n.s. | 0.10 | n.s. | -0.01 | n.s. | **-0.17** | **0.0464** |
| MaR1 | **-0.35** | **0.0262** | -0.06 | n.s. | 0.05 | n.s. | -0.08 | n.s. |
| MaR2 | -0.23 | n.s. | 0.04 | n.s. | 0.01 | n.s. | -0.05 | n.s. |
| NPD1 | 0.11 | n.s. | -0.01 | n.s. | 0.11 | n.s. | -0.07 | n.s. |
| RvE1 | -0.08 | n.s. | -0.02 | n.s. | 0.19 | n.s. | -0.06 | n.s. |
| RvE4 | 0.02 | n.s. | -0.25 | n.s. | 0.18 | n.s. | -0.09 | n.s. |
| LXA4 | 0.19 | n.s. | **-0.33** | **0.0304** | 0.20 | n.s. | -0.09 | n.s. |
| LTB4 | 0.09 | n.s. | -0.06 | n.s. | 0.09 | n.s. | 0.16 | n.s. |
| 14-HDHA | -0.20 | n.s. | 0.08 | n.s. | -0.01 | n.s. | -0.01 | n.s. |
| 17-HDHA | -0.07 | n.s. | -0.03 | n.s. | 0.15 | n.s. | 0.11 | n.s. |
| 20-HDHA | -0.06 | n.s. | 0.10 | n.s. | 0.11 | n.s. | 0.08 | n.s. |
| 12-HETE | -0.24 | n.s. | **-0.32** | **0.0340** | 0.08 | n.s. | -0.07 | n.s. |
| 15-HETE | -0.30 | n.s. | 0.03 | n.s. | -0.02 | n.s. | 0.03 | n.s. |
| PGD2 | 0.27 | n.s. | 0.03 | n.s. | 0.15 | n.s. | 0.04 | n.s. |
| PGE2 | -0.01 | n.s. | -0.23 | n.s. | 0.22 | n.s. | -0.09 | n.s. |
| PGF2a | 0.02 | n.s. | 0.14 | n.s. | 0.04 | n.s. | 0.00 | n.s. |
| DHA | -0.07 | n.s. | 0.04 | n.s. | 0.14 | n.s. | -0.06 | n.s. |
| EPA | -0.15 | n.s. | -0.12 | n.s. | 0.17 | n.s. | -0.07 | n.s. |
| AA | -0.26 | n.s. | 0.04 | n.s. | 0.13 | n.s. | 0.01 | n.s. |

| p-tau | AD | | MCI | | SCI | | All | |
| --- | --- | --- | --- | --- | --- | --- | --- | --- |
|  | r | P value | r | P value | r | P value | r | P value |
| RvD1 | -0.19 | n.s. | 0.00 | n.s. | 0.04 | n.s. | -0.13 | n.s. |
| RvD3 | 0.01 | n.s. | -0.19 | n.s. | 0.00 | n.s. | -0.01 | n.s. |
| RvD4 | -0.01 | n.s. | 0.15 | n.s. | 0.00 | n.s. | -0.12 | n.s. |
| MaR1 | -0.27 | n.s. | -0.01 | n.s. | 0.02 | n.s. | -0.08 | n.s. |
| MaR2 | -0.24 | n.s. | 0.04 | n.s. | -0.10 | n.s. | -0.10 | n.s. |
| NPD1 | 0.10 | n.s. | -0.08 | n.s. | 0.13 | n.s. | -0.06 | n.s. |
| RvE1 | -0.04 | n.s. | -0.02 | n.s. | 0.19 | n.s. | -0.05 | n.s. |
| RvE4 | 0.04 | n.s. | -0.21 | n.s. | 0.13 | n.s. | -0.07 | n.s. |
| LXA4 | 0.22 | n.s. | **-0.33** | **0.0306** | 0.23 | n.s. | -0.05 | n.s. |
| LTB4 | 0.05 | n.s. | -0.06 | n.s. | 0.16 | n.s. | 0.14 | n.s. |
| 14-HDHA | -0.18 | n.s. | 0.06 | n.s. | -0.05 | n.s. | 0.00 | n.s. |
| 17-HDHA | -0.07 | n.s. | -0.04 | n.s. | 0.12 | n.s. | 0.09 | n.s. |
| 20-HDHA | -0.05 | n.s. | 0.10 | n.s. | 0.13 | n.s. | 0.10 | n.s. |
| 12-HETE | -0.18 | n.s. | -0.28 | n.s. | 0.07 | n.s. | -0.08 | n.s. |
| 15-HETE | -0.27 | n.s. | 0.06 | n.s. | 0.03 | n.s. | 0.03 | n.s. |
| PGD2 | **0.32** | **0.0408** | 0.07 | n.s. | 0.15 | n.s. | 0.07 | n.s. |
| PGE2 | -0.02 | n.s. | -0.20 | n.s. | 0.20 | n.s. | -0.08 | n.s. |
| PGF2a | 0.02 | n.s. | 0.12 | n.s. | 0.00 | n.s. | -0.01 | n.s. |
| DHA | 0.02 | n.s. | 0.00 | n.s. | 0.06 | n.s. | -0.04 | n.s. |
| EPA | -0.05 | n.s. | -0.15 | n.s. | 0.14 | n.s. | -0.04 | n.s. |
| AA | -0.20 | n.s. | 0.02 | n.s. | 0.14 | n.s. | 0.02 | n.s. |

Correlations of lipid mediator (LM) levels in CSF samples of the entire cohort of individuals with Alzheimer's disease (AD), mild cognitive impairment (MCI) or subjective cognitive impairment (SCI), or all cases together (All) to the mini-mental state examination (MMSE) test scores and to levels of Aβ_42_, total (t)-tau, and phosphorylated (p)-tau. The data are presented by the r-value according to Spearman rank-order test together with the *P*-value, except when non-significant (n.s.). Significant correlations are presented in bold digits. AA = arachidonic acid, DHA = docosahexaenoic acid, EPA = eicosapentaenoic acid, HDHA = hydroxy-docosahexaenoic acids, HETE = hydroxy-eicosatetraenoic, LT = leukotriene, LX = lipoxin, MaR = maresin, NPD = neuroprotectin D, PG = prostaglandin, Rv = resolving

**Supplementary Table 3. LMs in CSF from patients diagnosed with AD, MCI, or SCI within the age-matched cohort.**

| **LM** | **AD** | | | **MCI** | | | **SCI** | | |
| --- | --- | --- | --- | --- | --- | --- | --- | --- | --- |
|  | Median | Lower Q | Upper Q | Median | Lower Q | Upper Q | Median | Lower Q | Upper Q |
| **RVD1** | 7.76 | *0.00* | *7.91* | 7.87 | *7.67* | *8.14* | 8.06 | *7.92* | *15.81* |
| **RVD3** | 0.76 | *0.09* | *0.97* | 0.68 | *0.22* | *0.90* | 0.79 | *0.67* | *1.81* |
| **RVD4** | 0.92 | *0.16* | *0.96* | 0.92 | *0.87* | *0.94* | 1.20 | *0.99* | *1.36* |
| **MaR1** | 0.18 | *0.05* | *0.26* | 0.18 | *0.12* | *0.21* | 0.26 | *0.19* | *0.34* |
| **MaR2** | 0.19 | *0.10* | *0.29* | 0.20 | *0.11* | *0.22* | 0.20 | *0.17* | *0.32* |
| **NPD1** | 0.35 | *0.25* | *0.41* | 0.31 | *0.24* | *0.36* | 0.38 | *0.25* | *0.43* |
| **RVE1** | 0.75 | *0.21* | *1.05* | 1.07 | *0.82* | *1.13* | 1.20 | *0.74* | *1.53* |
| **RVE4** | 1.39 | *0.50* | *1.69* | 1.15 | *0.43* | *1.30* | 1.49 | *1.32* | *2.23* |
| **LXA4** | 0.40 | *0.21* | *0.58* | 0.41 | *0.37* | *0.60* | 0.53 | *0.41* | *0.69* |
| **LTB4** | 0.42 | *0.33* | *0.65* | 0.45 | *0.31* | *0.51* | 0.33 | *0.25* | *0.40* |
| **14-HDHA** | 0.19 | *0.13* | *0.26* | 0.18 | *0.15* | *0.23* | 0.15 | *0.10* | *0.24* |
| **17-HDAH** | 0.21 | *0.14* | *0.29* | 0.18 | *0.12* | *0.23* | 0.16 | *0.10* | *0.21* |
| **20-HDHA** | 0.85 | *0.63* | *0.94* | 0.98 | *0.62* | *1.14* | 0.65 | *0.55* | *0.98* |
| **12-HETE** | 1.12 | *0.00* | *1.52* | 1.28 | *0.00* | *1.66* | 0.25 | *0.00* | *1.53* |
| **15-HETE** | 0.90 | *0.52* | *1.27* | 0.90 | *0.52* | *1.48* | 0.50 | *0.36* | *0.61* |
| **PGD2** | 22.68 | *19.88* | *24.47* | 20.29 | *19.49* | *21.74* | 23.62 | *18.67* | *33.88* |
| **PGE2** | 28.88 | *22.93* | *32.21* | 26.69 | *25.99* | *28.59* | 32.45 | *27.93* | *47.92* |
| **PGF2a** | 9.26 | *3.69* | *11.72* | 10.28 | *9.04* | *11.23* | 12.98 | *9.54* | *22.53* |
| **DHA** | 501.25 | *174.39* | *597.47* | 619.11 | *459.59* | *910.50* | 619.59 | *404.47* | *850.25* |
| **EPA** | 46.17 | *29.56* | *69.30* | 67.23 | *44.81* | *95.74* | 59.90 | *42.92* | *95.00* |
| **AA** | 124.84 | *108.83* | *152.37* | 167.45 | *99.52* | *221.95* | 117.48 | *105.24* | *202.35* |

The levels of lipid mediators (LMs) and polyunsaturated fatty acids (PUFAs) in cerebrospinal fluid (CSF) samples of the entire cohort of individuals with Alzheimer's disease (AD), mild cognitive impairment (MCI), and subjective cognitive impairment (SCI) are presented as median and interquartile (Q) range in fg/ml. AA = arachidonic acid, DHA = docosahexaenoic acid, EPA = eicosapentaenoic acid, HDHA = hydroxy-docosahexaenoic acids, HETE = hydroxy-eicosatetraenoic, LT = leukotriene, LX = lipoxin, MaR = maresin, NPD = neuroprotectin D, PG = prostaglandin, Rv = resolvin

**Supplementary Table 4a-d. Correlation of LMs to cognition and CSF plaque and tangle biomarkers (age-matched cohort).**

| **MMSE** | **AD** | | **MCI** | | **SCI** | | **All** | |
| --- | --- | --- | --- | --- | --- | --- | --- | --- |
|  | r | *P* value | r | *P* value | r | *P* value | r | *P* value |
| **RvD1** | -0.24 | n.s | -0.07 | n.s | 0.15 | n.s | 0.24 | n.s |
| **RvD3** | -0.03 | n.s | 0.03 | n.s | 0.20 | n.s | 0.21 | n.s |
| **RvD4** | -0.23 | n.s | -0.11 | n.s | **0.56** | **0.0088** | **0.39** | **0.044** |
| **MaR1** | -0.26 | n.s | -0.31 | n.s | 0.25 | n.s | 0.11 | n.s |
| **MaR2** | -0.22 | n.s | 0.03 | n.s | 0.13 | n.s | 0.12 | n.s |
| **NPD1** | -0.38 | n.s | 0.01 | n.s | 0.03 | n.s | -0.06 | n.s |
| **RvE1** | 0.01 | n.s | -0.11 | n.s | 0.36 | n.s | **0.34** | **0.0114** |
| **RvE4** | -0.17 | n.s | -0.23 | n.s | **0.48** | **0.0290** | 0.20 | n.s |
| **LXA4** | -0.33 | n.s | -0.29 | n.s | 0.38 | n.s | 0.18 | n.s |
| **LTB4** | 0.07 | n.s | **0.52** | **0.0319** | 0.24 | n.s | 0.02 | n.s |
| **14-HDHA** | **0.77** | **0.0008** | 0.15 | n.s | -0.04 | n.s | 0.06 | n.s |
| **17-HDHA** | 0.31 | n.s | 0.10 | n.s | 0.08 | n.s | -0.05 | n.s |
| **20-HDHA** | 0.24 | n.s | 0.29 | n.s | -0.17 | n.s | 0.07 | n.s |
| **12-HETE** | 0.11 | n.s | -0.05 | n.s | -0.13 | n.s | -0.03 | n.s |
| **15-HETE** | 0.26 | n.s | 0.13 | n.s | 0.10 | n.s | -0.06 | n.s |
| **PGD2** | -0.08 | n.s | 0.06 | n.s | -0.14 | n.s | -0.02 | n.s |
| **PGE2** | 0.29 | n.s | -0.02 | n.s | -0.23 | n.s | 0.06 | n.s |
| **PGF2a** | -0.04 | n.s | -0.10 | n.s | 0.15 | n.s | **0.30** | **0.0306** |
| **DHA** | **0.76** | **0.0011** | 0.18 | n.s | -0.03 | n.s | **0.34** | **0.0120** |
| **EPA** | 0.48 | n.s | 0.09 | n.s | -0.02 | n.s | 0.27 | n.s |
| **AA** | **0.62** | **0.0138** | 0.06 | n.s | -0.25 | n.s | 0.09 | n.s |

| **Aβ42** | **AD** | | **MCI** | | **SCI** | | **All** | |
| --- | --- | --- | --- | --- | --- | --- | --- | --- |
|  | r | *P* value | r | *P* value | r | *P* value | r | *P* value |
| **RvD1** | 0.03 | n.s | 0.06 | n.s | 0.03 | n.s | 0.25 | n.s |
| **RvD3** | 0.06 | n.s | 0.21 | n.s | -0.36 | n.s | 0.06 | n.s |
| **RvD4** | 0.36 | n.s | 0.39 | n.s | 0.40 | n.s | **0.46** | **0.0005** |
| **MaR1** | 0.10 | n.s | 0.28 | n.s | 0.01 | n.s | 0.20 | n.s |
| **MaR2** | 0.06 | n.s | -0.24 | n.s | **-0.68** | **0.0008** | -0.17 | n.s |
| **NPD1** | -0.37 | n.s | **-0.52** | **0.0307** | 0.11 | n.s | -0.14 | n.s |
| **RvE1** | -0.12 | n.s | 0.29 | n.s | 0.29 | n.s | **0.42** | **0.0016** |
| **RvE4** | 0.50 | n.s | -0.10 | n.s | 0.07 | n.s | 0.13 | n.s |
| **LXA4** | 0.45 | n.s | -0.24 | n.s | 0.07 | n.s | 0.20 | n.s |
| **LTB4** | **0.52** | **0.0462** | 0.08 | n.s | 0.20 | n.s | -0.03 | n.s |
| **14-HDHA** | **0.54** | **0.0365** | -0.13 | n.s | -0.27 | n.s | -0.12 | n.s |
| **17-HDHA** | 0.22 | n.s | -0.19 | n.s | -0.10 | n.s | -0.18 | n.s |
| **20-HDHA** | **0.59** | **0.0198** | 0.01 | n.s | 0.16 | n.s | 0.12 | n.s |
| **12-HETE** | **0.58** | **0.0221** | 0.04 | n.s | 0.06 | n.s | 0.15 | n.s |
| **15-HETE** | 0.30 | n.s | -0.25 | n.s | 0.03 | n.s | -0.12 | n.s |
| **PGD2** | -0.11 | n.s | -0.18 | n.s | -0.12 | n.s | -0.13 | n.s |
| **PGE2** | 0.00 | n.s | -0.48 | n.s | 0.03 | n.s | 0.06 | n.s |
| **PGF2a** | 0.42 | n.s | 0.13 | n.s | -0.05 | n.s | 0.25 | n.s |
| **DHA** | 0.30 | n.s | -0.01 | n.s | 0.09 | n.s | 0.26 | n.s |
| **EPA** | 0.37 | n.s | 0.10 | n.s | 0.03 | n.s | 0.24 | n.s |
| **AA** | **0.53** | **0.0428** | -0.09 | n.s | 0.14 | n.s | 0.18 | n.s |

| **t-tau** | **AD** | | **MCI** | | **SCI** | | **All** | |
| --- | --- | --- | --- | --- | --- | --- | --- | --- |
|  | r | *P* value | r | *P* value | r | *P* value | r | *P* value |
| **RvD1** | -0.50 | n.s | -0.24 | n.s | 0.07 | n.s | **-0.33** | **0.0154** |
| **RvD3** | -0.19 | n.s | -0.27 | n.s | -0.15 | n.s | -0.22 | n.s |
| **RvD4** | -0.37 | n.s | 0.06 | n.s | 0.16 | n.s | -0.24 | n.s |
| **MaR1** | -0.33 | n.s | -0.38 | n.s | -0.10 | n.s | **-0.33** | **0.0147** |
| **MaR2** | -0.33 | n.s | -0.17 | n.s | 0.04 | n.s | -0.11 | n.s |
| **NPD1** | 0.47 | n.s | 0.07 | n.s | 0.07 | n.s | 0.12 | n.s |
| **RvE1** | -0.03 | n.s | -0.46 | n.s | 0.06 | n.s | **-0.27** | **0.0468** |
| **RvE4** | -0.38 | n.s | -0.30 | n.s | **0.46** | **0.0360** | -0.10 | n.s |
| **LXA4** | 0.11 | n.s | -0.21 | n.s | 0.23 | n.s | -0.08 | n.s |
| **LTB4** | 0.26 | n.s | 0.17 | n.s | 0.23 | n.s | **0.36** | **0.0087** |
| **14-HDHA** | -0.26 | n.s | 0.08 | n.s | -0.08 | n.s | -0.04 | n.s |
| **17-HDHA** | **-0.56** | **0.0284** | -0.08 | n.s | 0.16 | n.s | 0.03 | n.s |
| **20-HDHA** | -0.40 | n.s | 0.01 | n.s | -0.20 | n.s | -0.08 | n.s |
| **12-HETE** | -0.51 | n.s | -0.36 | n.s | -0.37 | n.s | **-0.32** | **0.0195** |
| **15-HETE** | **-0.58** | **0.0238** | -0.36 | n.s | -0.09 | n.s | -0.08 | n.s |
| **PGD2** | **0.61** | **0.0148** | -0.12 | n.s | 0.28 | n.s | 0.15 | n.s |
| **PGE2** | 0.19 | n.s | -0.37 | n.s | 0.33 | n.s | -0.02 | n.s |
| **PGF2a** | -0.48 | n.s | -0.02 | n.s | 0.21 | n.s | -0.21 | n.s |
| **DHA** | **-0.71** | **0.0029** | -0.03 | n.s | -0.07 | n.s | **-0.33** | **0.0160** |
| **EPA** | **-0.81** | **0.0002** | -0.35 | n.s | -0.11 | n.s | **-0.40** | **0.0034** |
| **AA** | -0.29 | n.s | -0.11 | n.s | -0.10 | n.s | -0.18 | n.s |

| **p-tau** | **AD** | | **MCI** | | **SCI** | | **All** | |
| --- | --- | --- | --- | --- | --- | --- | --- | --- |
|  | r | *P* value | r | *P* value | r | *P* value | r | *P* value |
| **RvD1** | **-0.62** | **0.0133** | -0.19 | n.s | 0.12 | n.s | **-0.32** | **0.0210** |
| **RvD3** | -0.19 | n.s | -0.14 | n.s | -0.12 | n.s | -0.17 | n.s |
| **RvD4** | -0.24 | n.s | 0.16 | n.s | 0.18 | n.s | -0.17 | n.s |
| **MaR1** | -0.20 | n.s | -0.26 | n.s | -0.05 | n.s | **-0.27** | **0.0468** |
| **MaR2** | -0.24 | n.s | -0.18 | n.s | -0.03 | n.s | -0.11 | n.s |
| **NPD1** | 0.49 | n.s | -0.05 | n.s | 0.12 | n.s | 0.11 | n.s |
| **RvE1** | -0.02 | n.s | -0.38 | n.s | 0.09 | n.s | -0.24 | n.s |
| **RvE4** | -0.31 | n.s | -0.16 | n.s | 0.32 | n.s | -0.09 | n.s |
| **LXA4** | 0.25 | n.s | -0.27 | n.s | 0.26 | n.s | -0.05 | n.s |
| **LTB4** | 0.13 | n.s | 0.22 | n.s | 0.21 | n.s | **0.35** | **0.0107** |
| **14-HDHA** | -0.17 | n.s | 0.04 | n.s | -0.18 | n.s | -0.05 | n.s |
| **17-HDHA** | -0.45 | n.s | -0.06 | n.s | 0.20 | n.s | 0.02 | n.s |
| **20-HDHA** | -0.38 | n.s | 0.09 | n.s | -0.15 | n.s | -0.05 | n.s |
| **12-HETE** | -0.40 | n.s | -0.28 | n.s | **-0.47** | **0.0335** | **-0.32** | **0.0202** |
| **15-HETE** | **-0.55** | **0.0328** | -0.32 | n.s | -0.09 | n.s | -0.11 | n.s |
| **PGD2** | **0.83** | **0.0001** | -0.12 | n.s | 0.32 | n.s | 0.21 | n.s |
| **PGE2** | 0.26 | n.s | -0.43 | n.s | 0.38 | n.s | 0.00 | n.s |
| **PGF2a** | -0.38 | n.s | -0.03 | n.s | 0.11 | n.s | -0.22 | n.s |
| **DHA** | -0.51 | n.s | 0.01 | n.s | -0.22 | n.s | **-0.31** | **0.0243** |
| **EPA** | **-0.60** | **0.0175** | -0.28 | n.s | -0.21 | n.s | **-0.38** | **0.0049** |
| **AA** | -0.17 | n.s | -0.06 | n.s | -0.17 | n.s | -0.16 | n.s |

Correlations of lipid mediator (LM) levels in CSF samples of the age-matched cohort of individuals with Alzheimer's disease (AD), mild cognitive impairment (MCI) or subjective cognitive impairment (SCI), or all cases together (All) to the mini-mental state examination (MMSE) test scores and to levels of Aβ_42_, total (t)-tau, and phosphorylated (p)-tau. The data are presented by the r-value according to Spearman rank-order test together with the *P*-value, except when non-significant (n.s.). Significant correlations are presented in bold digits. AA = arachidonic acid, DHA = docosahexaenoic acid, EPA = eicosapentaenoic acid, HDHA = hydroxy-docosahexaenoic acids, HETE = hydroxy-eicosatetraenoic, LT = leukotriene, LX = lipoxin, MaR = maresin, NPD = neuroprotectin D, PG = prostaglandin, Rv = resolvin
